# Current status of targeted microbubbles in diagnostic molecular imaging of pancreatic cancer

**DOI:** 10.1002/btm2.10183

**Published:** 2020-09-07

**Authors:** Natacha Jugniot, Rakesh Bam, Emmanuelle J. Meuillet, Evan C. Unger, Ramasamy Paulmurugan

**Affiliations:** ^1^ Department of Radiology Molecular Imaging Program at Stanford, Stanford University Palo Alto California USA; ^2^ NuvOx Pharma Tucson Arizona USA

**Keywords:** cancer early detection, clinical translation, microbubbles, molecular imaging, pancreatic ductal adenocarcinoma, targeted‐imaging agents, ultrasound

## Abstract

Pancreatic ductal adenocarcinoma (PDAC) is often associated with a poor prognosis due to silent onset, resistance to therapies, and rapid spreading. Most patients are ineligible for curable surgery as they present with advanced disease at the time of diagnosis. Present diagnostic methods relying on anatomical changes have various limitations including difficulty to discriminate between benign and malignant conditions, invasiveness, the ambiguity of imaging results, or the inability to detect molecular biomarkers of PDAC initiation and progression. Therefore, new imaging technologies with high sensitivity and specificity are critically needed for accurately detecting PDAC and noninvasively characterizing molecular features driving its pathogenesis. Contrast enhanced targeted ultrasound (CETUS) is an upcoming molecular imaging modality that specifically addresses these issues. Unlike anatomical imaging modalities such as CT and MRI, molecular imaging using CETUS is promising for early and accurate detection of PDAC. The use of molecularly targeted microbubbles that bind to neovascular targets can enhance the ultrasound signal specifically from malignant PDAC tissues. This review discusses the current state of diagnostic imaging modalities for pancreatic cancer and places a special focus on ultrasound targeted‐microbubble technology together with its clinical translatability for PDAC detection.

## 1 INTRODUCTION

Pancreatic ductal adenocarcinoma (PDAC) has one of the worst prognoses of all types of cancer, and its rising incidence projects the disease to become the second deadliest cancer by 2030 after lung cancer.^1^ Approximately 80% of patients with PDAC present with locally advanced or metastatic disease at their initial diagnosis, or do not qualify for a complete tumor resection due to its diffused nature. All tumor stages combined, PDAC has a dismal prognosis with a 5‐year survival rate of <9%.^2^ Even for the small percentage of patients diagnosed with localized disease, the 5‐year survival rate is only of 37%. The need for effective screening methods is globally recognized since diagnosing pancreatic diseases at an early stage (for example by serendipitous discovery in asymptomatic patients when evaluating unrelated disease) can drastically improve outcomes by providing patients opportunities for effective treatments with fewer complications.^3^

The detection of pancreatic cancer biomarkers in plasma or serum (e.g., proteins, circulating tumor cells, circulating nucleic acids, aberrantly expressed cancer‐associated antigens, metabolites, small molecules, and exosomes) appears promising.^4^, ^5^, ^6^ Nevertheless, serum carbohydrate antigen 19‐9 (CA 19‐9) is, for now, the only serum biomarker that has been routinely used in clinical practice to monitor PDAC progression, recurrence, and therapy response.^7^ Of high interest, serum CA 19‐9 level has been shown to be significantly upregulated as early as 2 years before pancreatic cancer diagnosis.^8^ Unfortunately, the reliability of CA 19‐9 as a biomarker has been compromised by false negative results in patients lacking fucosyltransferase activity.^9^ Moreover, false positive results are also observed in benign pancreaticobiliary diseases such as hepatic cyst,^10^ obstructive jaundice,^11^ cholangitis,^12^ and pancreatitis.^13^ Therefore, the accuracy of CA19‐9 is debatable, and it is recommended to diagnose patients using imaging techniques.^14^ For these reasons, imaging modalities that can detect PDAC with high sensitivity and specificity are under vigorous investigations.

Present imaging modalities utilized for suspected pancreatic cancer and for screening high‐risk patients include computed tomography (CT), magnetic resonance imaging (MRI), transabdominal ultrasound (US), endoscopic ultrasound (EUS) imaging, and positron emission tomography (PET) imaging. Among these, CT, MRI, and ultrasound imaging are anatomical imaging modalities. Molecular imaging techniques such as PET can complement those modalities by providing functional and molecular information.^15^ Contrast‐enhanced ultrasound (CEUS), MRI, PET, and fluorescence imaging (FI) are promising PDAC molecular imaging modalities. The accuracy of pancreatic cancer detection, especially during the disease initiation stages, is highly dependent on: (a) the specificity of the targeted biomarker, (b) the physical, biochemical and pharmacological characteristics of the contrast agent, and (c) the efficiency of imaging instrumentation and protocol. In this context, a promising technology nearing clinical translation strategizes the use of transabdominal ultrasound in combination with molecularly targeted microparticles, named microbubbles (MBs), that bind to neovascular targets within PDAC lesions and enhance the contrast in the imaging signal. Recently, this technology was evaluated for the neovascular target protein, Thy1, in mouse models of PDAC.^16^ This review brings attention to the current status of the targeted microbubble technology for PDAC diagnosis via ultrasound molecular imaging. We specifically discuss the details of this technology and compare it to alternative molecular imaging strategies, with a special emphasis on the sensitivity and specificity of these imaging modalities in differentiating PDAC from benign conditions. Finally, we culminate this review by providing an outlook toward clinical translation of ultrasound targeted PDAC contrast agents.

## 2 CURRENT STATUS OF PDAC DIAGNOSTIC IMAGING IN THE CLINIC

Over the past decade, multiple publications have reviewed the accuracy of the current clinical imaging modalities (CT, MRI, US, and PET) in detecting pancreatic cancers.^15^, ^17^, ^18^, ^19^ These modalities are briefly presented below under two different categories such as, anatomical imaging modalities, and molecular/metabolic imaging modalities. Main characteristics of those imaging modalities are summarized in Table 1.

**TABLE 1:** Clinical imaging modalities of pancreatic cancer

Imaging modality	Sensitivity (%)	Specificity (%)	Acquisition time	Invasiveness or radiation	Operator dependency	Depth	FOV	Widely available	Cost	Frequency of use
EUS‐FNA	85–92	95–98	Minutes to hour	Fine‐needle, endoscope, sedation/anesthesia	High	cm	Limited	No	++	+
CT (MDCT)	76–94	70–96	Minutes	IV contrast agent, radiation	Low	No limit	Body coverage	Yes	++	++++
MRI (MRCP)	85–93	72–79	Minutes to hour	IV contrast agent	Low	No limit	Body coverage	No	+++	+++
Transabdominal US	50–90	90–99	Minutes	No	High	cm	Limited	Yes	+	++
PET/CT	85–96	61–94	Minutes to hour	IV contrast agent, radiation	Low	No limit	Body coverage	No	++++	+

Abbreviations: CT, computed tomography; EUS, endoscopic ultrasound; FNA, fine needle aspiration; FOV, field of view; IV, intravenous; MDCT, multi‐detector computed tomography; MRI, magnetic resonance imaging; PET, positron emission tomography; + indicates relative cost or clinical frequency of use for pancreatic cancer imaging.

### 2.1 Anatomical imaging modalities

Anatomical imaging modalities provide useful information by detecting primary tumor or metastatic foci and aid in determining tumor resectability. Such imaging modalities exploit the differences between normal and abnormal tissue properties to create contrast. Transabdominal ultrasound, endoscopic ultrasound, CT and MRI, are some primary imaging modalities falling under this category.

#### 2.1.1 Transabdominal ultrasound

Transabdominal ultrasound imaging uses sound waves to produce pictures of the structures within the upper abdomen. It involves an ultrasound transducer pressed against the skin of the abdomen. Sound waves delivered from the transducer at a specific frequency bounce off tissues and create echoes. A computer, connected to the transducer, utilizes those sound waves to create an image (sonogram). For patients presenting with jaundice or abdominal pain, noninvasive transabdominal ultrasound is a frequently recommended initial imaging modality. Transabdominal ultrasound imaging is convenient, devoid of ionizing radiation, portable, widely available and economical. On the contrary, ultrasound imaging is a highly operator‐dependent modality, that sometimes cannot allow a panoramic view of the pancreas due to the presence of gas in the stomach or duodenum. As a direct consequence, the sensitivity of transabdominal ultrasound for detecting pancreatic cancer varies widely, from 50% (tumor <1 cm in diameter) to 90% (tumor >3 cm in diameter).^20^, ^21^ Moreover, differentiating pancreatic cancer from other focal lesions, such as chronic pancreatitis, is very challenging due to similarities in their superficial imaging features.^21^

#### 2.1.2 Endoscopic ultrasound

In EUS, ultrasound transducer is placed much closer to the pancreas than in transabdominal ultrasound. The ultrasound transducer is inserted into the mouth and positioned down into the first part of the small intestine. EUS imaging represents one of the most sensitive methods for detecting PDAC. It is especially useful to detect small tumors that are not visualized by other imaging modalities (typically <2 cm).^22^, ^23^ EUS also provides the opportunity to collect tissue samples by fine needle aspiration (FNA) of suspected pancreatic lesion during imaging session. Samples can be used for biochemical, cytologic, and/or DNA analysis, thus helping to confirm the diagnosis or to further characterize the tumor. However, the performance of traditional EUS can be limited, especially when imaging patients with symptomatic or asymptomatic inflammatory status, the inflammation can interfere with pancreatic cancer diagnosis.^24^ EUS interpretation relies on the normal pancreatic parenchyma as point of distinction to pathological lesions, typically hypoechoic. Acute pancreatitis can mask significant findings or be misinterpreted as a mass, and chronic pancreatitis can result in an extensive loss of normal pancreatic parenchyma. For those patients, EUS has a sensitivity and a specificity of 63.6 and 75.9%, respectively.^25^ EUS‐FNA constitutes a more accurate diagnostic tool. A meta‐analysis evaluates the accuracy of EUS‐FNA with 86.9% sensitivity and 95.9% specificity.^26^ Nevertheless, to reach high sensitivity in patients with chronic pancreatitis is still a challenging task.^17^, ^27^, ^28^ Furthermore, EUS‐FNA is an operator dependent technique that requires sedation or sometimes general anesthesia, which may induce risks in some patients (e.g., cognitive dysfunction, malignant hyperthermia, breathing problems).^29^, ^30^ Although endoscopic ultrasound has high accuracy for the detection of small pancreatic tumors, it is invasive and considered as a complementary tool in current clinical practice.

#### 2.1.3 Computed tomography

Multi‐detector computed tomography (MDCT) is the most readily available imaging modality and the most frequently used technique in the diagnosis and staging of PDAC. It is also the gold standard to evaluate pancreatic resectability. For optimal performance, CT examination is performed with an intravenous iodinated contrast agent injection. CT has an overall sensitivity between 76 and 94% for diagnosing PDAC with a specificity between 70 and 96%.^31^, ^32^, ^33^ However, the sensitivity can decrease to 70% for smaller tumors (<2 cm), and can be as low as 67% for lesions <1.5 cm.^31^, ^34^ Thus, MDCT is inefficient in detecting small pancreatic lesions (<1 cm). Yet, as pancreatic cancer can start metastasize when tumors are <1 cm, detecting lesions at the earliest stage is of great importance.^35^ Additionally, current CT techniques cannot differentiate malignant and benign lesions.^17^, ^19^ Furthermore, CT is accompanied by the risk of nephrotoxicity from the injected iodine contrast agent, and capable of causing DNA damage from the ionizing radiations.

#### 2.1.4 Magnetic resonance imaging

MRI is another important imaging modality that can help in diagnosing patients at initial presentation. Usually, magnetic resonance is reserved as a second‐line imaging modality when suspected pancreatic tumors are not visible on CT or in case of equivocal CT findings. Because of its high soft tissue contrast, MRI has been preferred over CT for assessing small tumors in the pancreas, and for precisely detecting enlarged lymph nodes and distant metastases.^36^, ^37^ Magnetic resonance cholangiopancreatography (MRCP) is often included in the MRI examination to improve evaluation of the biliary and pancreatic ductal system. The lack of ionizing radiation for image acquisition makes MRI an ideal tool for follow‐up examinations. The sensitivity of MRI is low but can be improved by contrast agents modifying the T1 or T2 relaxation time constants of tissues (small gadolinium‐containing contrast agents and superparamagnetic iron oxide nanoparticles, respectively). MRI has a reported sensitivity and specificity for pancreatic cancer diagnosis ranging from 85 to 93% and from 72 to 79%, respectively.^38^, ^39^ Nevertheless, chronic pancreatitis and autoimmune pancreatitis can appear as focal mass mimicking pancreatic adenocarcinoma.^31^ Drawbacks of MRI include the susceptibility of the image quality to internal and external motions, long acquisition times, relatively high costs, lower availability relative to ultrasound and CT imaging, and potential contraindications (e.g., cardiac implantable electronic devices, metallic foreign bodies, implantable neurostimulation systems, drug infusion pumps, magnetic dental implants).

### 2.2 Metabolic imaging modalities

Compared to anatomical imaging modalities that are offering morphological information, PET provides exclusive information about the molecular and metabolic changes associated with the disease. This modality uses the metabolic radiotracer ^18^F‐fluoro‐2‐deoxy‐D‐glucose (^18^F‐FDG) that accumulates in cells with increased glycolytic metabolism. The high glycolytic rate in malignant cells forms the basis of PET imaging. Studies have reported its relatively high sensitivity and specificity in the detection of pancreatic malignancy, ranging from 85 to 96% and 61 to 94%, respectively.^38^, ^40^, ^41^, ^42^ Several studies have evaluated the ability to differentiate chronic pancreatitis from pancreatic cancer based on accumulation patterns of ^18^F‐FDG within the pancreas.^43^, ^44^, ^45^ Those studies have shown that the accumulation of ^18^F‐FDG in chronic pancreatitis generally demonstrated to be at low level with diffused ^18^F‐FDG uptake pattern compared to pancreatic cancer. However, ^18^F‐FDG lacks target‐related specificity, and active metabolic uptake, that is, positive results, can also be found in patients with benign conditions.^46^ New PET radiotracers with high specificity to differentiate pancreatic lesions with different level of malignancies are under development.^47^, ^48^

Globally, MDCT constitutes the first imaging modality in suspected pancreatic cancer patients. Alternatively, MRCP may be used in centers where this facility is readily available. PET‐CT could serve as a useful functional imaging approach. Finally, ultrasonography is an easy and fast way to image the pancreas area of patients with upper gastrointestinal complaints. EUS appears complementary to CT and MRI in the assessment of lesions not clearly detected, but suspected, on CT/MRI.^22^, ^23^, ^32^, ^49^ Despite the quality of those clinical imaging modalities, their accuracy is imperfect. Results are often inconclusive or clinically ambiguous. Particularly, patients with benign conditions like pancreatitis may present with images difficult to distinguish from PDAC.^50^ This is further confounding since patients with chronic pancreatitis are at increased risk of developing pancreatic cancer later, and patients with pancreatic cancer can have chronic pancreatitis by way of tumor related duct obstruction.^51^ The next big challenge is to accurately and noninvasively differentiate carcinoma from chronic pancreatitis or benign lesions presenting as PDAC in current imaging methods. To overcome these issues, identification of biomarkers that are differentially expressed by tumor cells or tumor‐associated vascular linings compared to benign or normal pancreatic tissues is needed as well as development of contrast agents to improve tumor contrast in relation to surrounding parenchyma.

### 2.3 Clinically approved ultrasound contrast agents

Ultrasound imaging could be a valuable diagnostic tool when a whole‐body imaging is not required. To improve the quality and the reliability of ultrasound scans, various methods are currently available. Among which contrast‐enhanced endoscopic ultrasound (CE‐EUS), endoscopic ultrasound elastography, and CEUS are being frequently employed in the clinic.

#### 2.3.1 Ultrasound imaging modality generalities

EUS elastography for the evaluation of the pancreatic tissues was first reported in 2006.^52^ EUS elastography quantifies elastic properties of pancreatic tissue in a real time manner. The incorporation of elastography into the evaluation of solid pancreatic lesions has resulted in a mean enhanced sensitivity of 93% with a specificity of 67%.^53^ To date, there are no medical reports available on EUS elastography for the diagnosis of cystic pancreatic lesions nor for staging pancreatic cancer. CE‐EUS and CEUS are two outstanding techniques using microbubbles as ultrasound contrast agents (UCAs). Microbubbles are gas‐filled microparticles of approximately 2–6 μm in diameter, and are pure vascular contrast agents. Their inert gas core can be air but most frequently heavier gases such as perfluorocarbon with low diffusion constants and low solubility in blood have been used. The outer shell of the contrast agents can consist of a variety of materials including albumin, polymers or phospholipids.^54^ Additionally, microbubbles can also be exploited to carry molecules, and have important applications as transporters of therapeutic drugs or genes.^55^ Microbubbles have the possibility to be conjugated to targeting ligands, referred as targeted‐microbubbles. By contrast, microbubbles without molecular targeting capacities are referred as nontargeted microbubbles. Microbubbles are highly echogenic in response to an incident ultrasound beam due to the impedance mismatch against the surrounding tissue.^56^ They can oscillate nonlinearly upon interacting with the ultrasound wave and provide greater tissue contrast in relation to the background signal.^57^ The desmoplastic reaction and the increase in extracellular matrix deposition elevate PDAC tumor interstitial fluid pressure and blood vessel compression, which reduces blood flow and decreased perfusion of UCAs. UCAs show homogeneous contrast enhancement in normal pancreatic parenchyma, whereas pancreatic adenocarcinoma shows hypoenhancement. Reprogramming PDAC desmoplastic stroma through the activation of vitamin D receptor signaling in cancer‐associated fibroblasts,^58^ gold‐nanoparticle‐induced alterations in PDAC secretome,^59^ and extracellular‐matrix modifying agents targeted to hyaluronan^60^ promote angiogenesis, decrease tumor stiffness and increase perfusion. Such treatment approaches may transform PDAC vascularity in humans, which in turn will aid in increasing not only the delivery of therapeutics but also the sensitivity of CETUS imaging. However, increasing angiogenesis is a double‐edged sword and must be carefully controlled since it promotes tumor growth and metastasis.^61^ This is the reason why many therapeutic strategies either involve the inhibition of pro‐angiogenic factors (e.g., vascular endothelial growth factor [VEGF]), either use endogenous angiogenesis inhibitors such as endostatin or angiostatin. A meta‐analysis has shown that CE‐EUS has a sensitivity of 92% and a specificity of 86% in the diagnosis of pancreatic tumors.^62^ On the other hand, CEUS has proved to provide tissue information with both high specificity and sensitivity. Two meta‐analysis have been published with pooled sensitivity and specificity of 93–94% and 88–89%, respectively.^63^, ^64^ Compared to CE‐EUS, CEUS has the main advantage to work without endoscopy for similar sensitivity and specificity, and thus is often preferred. Details on microbubbles used in clinics for CEUS imaging of pancreatic cancer are presented below.

#### 2.3.2 FDA‐approved ultrasound contrast agents

CEUS is a well‐established modality with official guidelines and recommendations available for clinical applications.^65^ Guidelines for the use of CEUS for pancreatic lesions were introduced in 2011 by the European Federation of Societies for Ultrasound in Medicine and Biology (EFSUMB), and World Federation for Ultrasound in Medicine and Biology.^66^, ^67^ In the United States, the Food and Drug Administration (FDA) has approved several nontargeted microbubbles to improve organ imaging and assess tissue perfusion.^68^ CEUS using microbubbles significantly improves blood flow visualization and inform about macro‐ and microvascularity, and has common application in vascular disease imaging.

A representative list of clinically employed microbubbles includes Optison (GE Healthcare, Princeton, NJ), Levovist (Schering AG, Berlin, Germany), Echovist (Schering AG, Berlin, Germany), Imagent (Imavist) (Schering AG, Berlin, Germany), SonoVue (Lumason) (BRACCO, Geneva, Switzerland), Definity (Luminity) (Lantheus Medical Imaging, N. Billerica, MA), Albunex (Molecular Biosytems Inc., San Diego, CA), and Sonazoid (GE Healthcare, Olso, Norway) (Table 2). The gas contained in the first‐generation microbubbles, such as Albunex, Levovist and Echovist, included air with poor duration of ultrasound enhancement due to rapid diffusion out of the microbubbles into the blood. The second‐generation utilized inert and heavy gases with low diffusion coefficient such as sulfur hexafluoride or perfluorobutane (Imagent, SonoVue, Definity, Optison and Sonazoid).^69^ However, currently used microbubbles acknowledge limitation with their wide size distribution range, which needs optimization according to the transducer frequency bandwidth to increase imaging efficiency. Nevertheless, these microbubbles provided the basis for the design and synthesis of molecularly targeted UCAs, ultimately contributing to the development of ultrasound molecular imaging techniques for accurate and early detection of PDAC.

**TABLE 2:** Overview of FDA approved ultrasound contrast agents compared to molecularly targeted BR55 and MB_Thy1‐scFv_ for PDAC detection

Name	Year	Shell component(s)	Gas component(s)	Mean diameter (μm)	Molecular target	Applications	Concentration (10^9^/ml)	Approval status	Producer/distributor
Echovist	1991	Galactose‐based shell	Air	99% <12	None	Heart imaging	—	FDA‐approved	Schering AG, Berlin, Germany
Albunex	1993	Albumin shell	Air	4 ± 2	None	Lung imaging	0.5–0.8	FDA‐approved	Molecular Biosystems Inc., San Diego, CA
Levovist	1995	Galactose‐based shell	Air	95% <10	None	Heart imaging	—	FDA‐approved	Schering AG, Berlin, Germany
Optison	1898	Albumin shell	Octafluoropropane	2–4.5	None	Heart imaging	0.5–0.8	FDA‐approved	GE Healthcare, Princeton, NJ
Definity (Luminity)	2001/2006	Phospholipid shell, DPPA, DPPC, MPEG5000DPPE	Octafluoropropane	2.5	None	Heart imaging	1–1.5	FDA‐approved	Lantheus Medical Imaging, North Billerica, MASA
SonoVue (Lumason)	2001/2014	Phospholipid shell: DSPC, DPPG, palmitic acid, PEG4000	Sulfurhexafluoride	2.5	None	Heart imaging, liver lesions, breast lesions	0.1–0.5	FDA‐approved	Bracco, Geneva, Switzerland
Imagent (Imavist)	2002	Phospholipid shell: DMPC	Perfluorohexane, nitrogen	5	None	Heart imaging	0.5	FDA‐approved	Schering AG, Berlin, Germany
Sonozoid	2007	Phospholipid shell	Perfluorobutane	3	None	Heart imaging, liver imaging	1	FDA‐submitted	GE Healthcare, Oslo, Norway
BR55	2015	Phospholipid shell: DSPE‐PEG(2000)‐peptide	Perfluorobutane, nitrogen	1.5 ± 0.1	VEGFR2	Prostate cancer imaging, ovarian cancer imaging, pancreatic cancer imaging	2	Phases 1 and 2 clinical trials	Bracco, Geneva, Switzerland
MB_Thy1‐scFv_	2020	Phospholipid shell: DPPC, DPPE, DPPE‐PEG(5000)‐MPEG, DSPE‐PEG(5000)‐MA	Octafluoropropane	2 ± 0.41	Thy1	Pancreatic cancer imaging	0.5	Preclinical assay	16

Abbreviations: DMPC, 1,2‐dimyristoyl‐sn‐glycero‐3‐phosphocholine; DPPA, 1,2‐Dipalmitoyl‐sn‐glycero‐3‐phosphatidic acid; DPPC, 1,2‐dipalmitoylphosphatidylcholine; DPPE, 1,2‐Dipalmitoyl‐snglycero‐3‐phosphoethanolamine; DPPE‐PEG(5000)‐MPEG, 1,2‐Dipalmitoyl‐sn‐glycero‐3‐phosphoethanolamine‐*N*‐ carbonyl‐methoxypolyethyleneglycol(polyethylene glycol)‐5000; DPPG, 1,2‐Dipalmitoyl‐sn‐glycero‐3‐phosphorylglycerol; DSPC, 1,2‐distearoyl‐sn‐glycero‐3‐phosphocholine; DSPE‐PEG(2000), 1,2‐distearoyl‐sn‐glycero‐3‐phosphoethanolamine‐*N*‐amino(polyethylene glycol)‐2000; DSPE‐PEG(5000)‐MA, 1,2‐distearoyl‐sn‐glycero‐3‐phosphoethanolamine‐*N*‐maleimide(polyethylene glycol)‐5000; food and drug administration; MPEG5000DPPE, *N*‐(Carbonyl‐methoxypolyethyleneglycol 5000)‐1,2‐Dipalmitoyl‐sn‐glycero‐3‐phosphoethanolamine; PEG4000, Polyethylene glycol‐4000; scFv, single‐chain variable fragment; Thyl, thymocyte differentiation antigen 1; VEGFR2, vascular endothelial growth factor receptor 2.

### 2.4 Molecularly targeted UCAs in PDAC imaging

Molecularly targeted ultrasound imaging relies on systemically delivering contrast agents that bind to biological markers overexpressed in vascular margins of the pathological tissues. Contrary to nontargeted UCAs, targeted UCAs have the great potential to improve pancreatic cancer early detection imaging with greater sensitivity and specificity.

Targeted microbubbles are synthesized by conjugating targeting ligands to the bubble shell during their formation. Alternatively, ligands can be conjugated to one of the shell components (e.g., a phospholipid species) prior to bubble formation. The ability of microbubbles to bind to a particular target in vivo and to accumulate at the site of the disease, largely depends on the abundance of the target molecules in the tissue, the properties of the targeting ligand and their density on the bubble surface, the bubble characteristic, dose, and the imaging protocol employed.^70^

Several research groups have focused on developing microbubbles targeting specific vascular endothelial biomarkers in pancreatic cancer.^16^, ^71^, ^72^, ^73^, ^74^ A recently published article describing a new strategy for targeted microbubble appears to be of high interest for PDAC ultrasound molecular imaging in clinical settings. This section of the review outlines strengths and limitations of the targeted microbubble technology^16^ and discuss possible alternatives.

#### 2.4.1 Overview of MB_Thy1‐scFv_ technology

**FIGURE 1:**
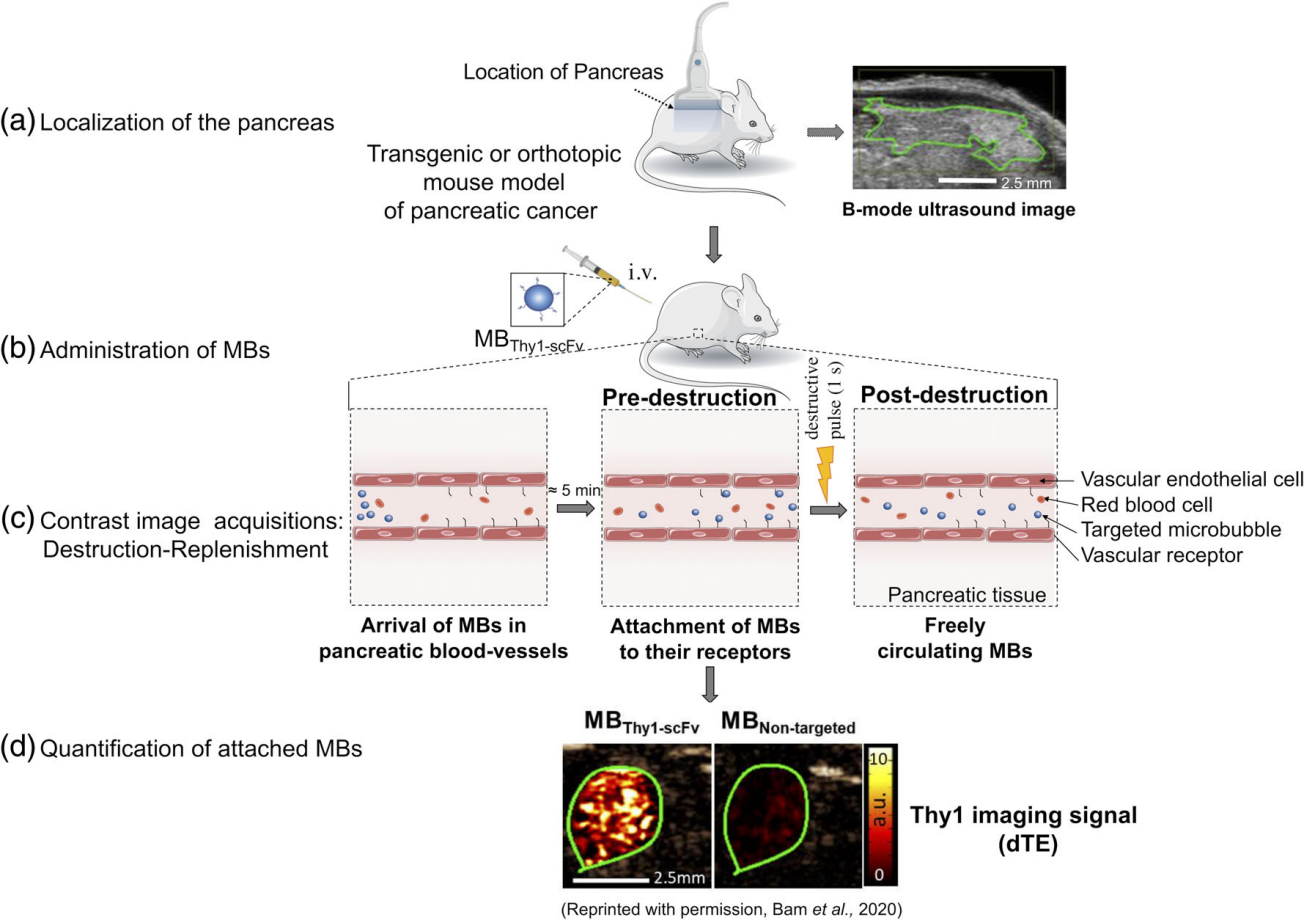
Molecularly‐targeted microbubble imaging in mouse. (a), Targeted microbubbles are administrated intravenously via the tail vein of pancreatic cancer mouse models. (b), An ultrasound linear transducer is placed over the abdomen of the mouse and the pancreas is localized. (c), Contrast mode images are acquired starting 4 min after the administration of the microbubbles to allow their attachment to their targets. (d), The application of a high‐pressure destructive pulse allows the quantification of blood‐vessel attached contrast agents. The difference in ultrasound signal pre‐ and postdestruction corresponds to the molecular signal from attached MB_Thy1‐scFv_ or MB_Nontargeted._ dTE, differential targeted enhancement; IV, intravenous; MB, microbubble

**FIGURE 2:**
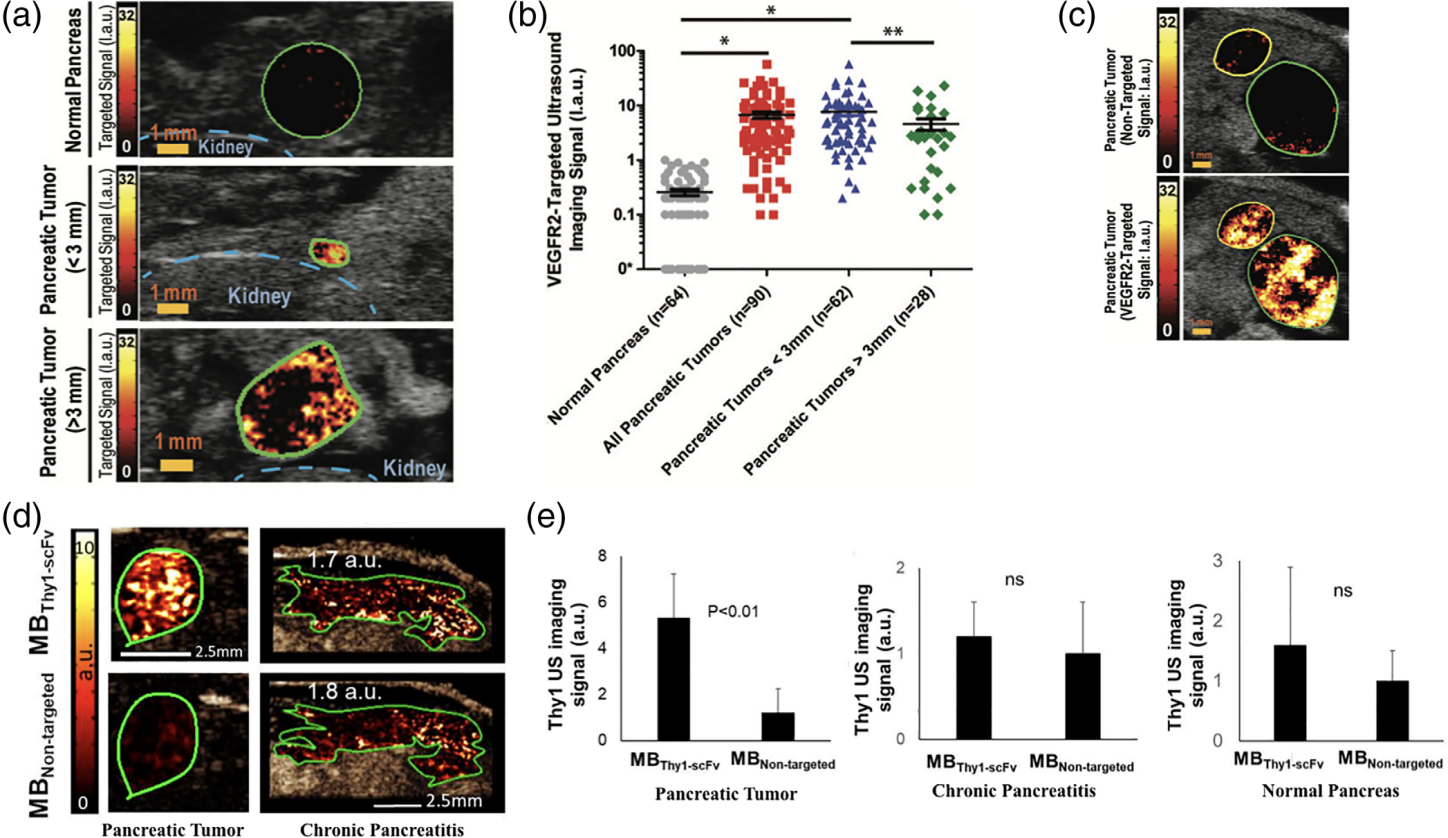
The use of microbubbles in molecular imaging of pancreatic cancer in mouse models. (a), VEGFR2‐targeted US signal is shown in normal pancreas and two foci of PDAC in two different transgenic mice. The outline of the kidney is shown in blue. (b), Dot plot summarizes mean VEGFR2‐targeted US signal measured in normal pancreas (*n* = 64) and all pancreatic tumors (*n* = 90). Pancreatic tumors were further analyzed by tumor diameter—smaller than 3 mm (*n* = 62) and larger than 3 mm (*n* = 28). (c), Representative transverse targeted US images show two adjacent foci of PDAC, imaged sequentially with nontargeted microbubbles and VEGFR2‐targeted microbubbles. (d), in vivo ultrasonographic molecular imaging of a pancreatic tumor, chronic pancreatitis (induced in mice by subcutaneous injection of l‐arginine) after intravenous injection of Thy1‐targeted microbubbles (MB_Thy1‐scFv_) and untargeted microbubbles (MB_nontargeted_). (e), Bar graph of Thy1‐specific ultrasound molecular signal in murine pancreatic tumor, chronic pancreatitis and normal pancreas. Color‐coded scale is shown for ultrasonographic molecular imaging signal in arbitrary units (a.u.). Green and yellow lines represent principal and secondary regions of interest, respectively. Error bars represent standard deviation. * = *p* < .001, ** = *p* < .024. Permission obtained from Radiology RSNA, Pysz MA., et al. *Radiology*. 2015;274:790‐799 (a‐c)^73^ and from Bam R, et al. *Invest Radiol*. Accepted article. 2020 (c & e)^16^

Bam et al., engineered a clinically translatable Thy1‐targeted microbubble (MB_Thy1‐scFv_) as UCA. It specifically recognizes the thymocyte differentiation antigen (Thy1/CD90) overexpressed on the surface of vascular endothelial cells of PDAC tissues compared to normal and inflamed tissues.^75^, ^76^ Thy1 has been previously validated as a clinically relevant biomarker for ultrasound imaging of PDAC.^71^ MB_Thy1‐scFv_ consists of a gas core of octafluoropropane surrounded by a phospholipid shell, with a mean diameter of 2.0 ± 0.41 μm. To specifically target Thy1 protein, a single‐chain antibody fragment (scFv) was engineered and conjugated to the microbubbles by maleimide‐thiol chemistry. Targeted microbubbles were intravenously administrated in mice and applied for CETUS imaging (Figure 1). MB_Thy1‐scFv_ was tested in several mouse models including a transgenic mouse model of PDAC, mice with l‐arginine‐induced pancreatitis and mice with healthy pancreas. MB_Thy1‐scFv_ produced higher ultrasound signal (5.3 ± 1.9 a.u.) from tumor tissues compared to the nontargeted microbubbles (MB_Nontargeted_) (1.2 ± 1.0 a.u.) in mice with PDAC. Importantly, in vivo results demonstrated the advantage of MB_Thy1‐scFv_ in differentiating PDAC from pancreatitis and from normal pancreatic tissue. Imaging signals in both normal and inflamed tissues with MB_Thy1‐scFv_ or MB_Nontargeted_ contrast agents were not statistically different, and were significantly lower than the molecular imaging signal with MB_Thy1‐scFv_ in PDAC tumors (Figure 2). Those results confirmed the ability of MB_Thy1‐scFv_ to specifically bind Thy1 in PDAC tissues and to significantly enhance ultrasound contrast signal. To confirm clinical feasibility of this technology, Bam et al. also tested the binding specificity of scFv ligand to Thy1 on human PDAC tissue samples. Using immunofluorescence staining, they validated Thy1‐scFv binding to VEGFR2‐positive vasculature and fibroblasts in tissue samples of various PDAC grades. Overall, these results showed the clinical potential of Thy1‐targeted microbubbles for a noninvasive and accurate detection of small tumors, based on molecular features that distinguish PDAC from benign conditions such as pancreatitis.

Ongoing research projects for complete clinical application of targeted microbubbles explore other signal quantification methods, which are compatible for human testing, faster to analyze, and more real‐time.^77^ Contrary to most other imaging techniques like PET/CT that frequently require more than 1 hr between the imaging agent injection and data acquisition, ultrasound imaging using targeted microbubbles could be performed a few minutes following intravenous administration of the contrast agents in patients. Such property would thus allow multiple injections of targeted microbubbles within the same imaging session. While microbubbles are useful blood pool contrast agents for imaging the tumor endothelium, imaging the cancer cells requires a different strategy for ultrasound molecular imaging of PDAC. Typically, tumor vessels are permeable to particles smaller than 1 μm. Preclinical development of nano‐size UCAs are in process in the aim to widen the possible tumor specific biomarker targets located beyond the vasculature. Thus, nano‐size UCAs have the added value over microbubbles in directly imaging the extravascular space of the tumor possibly via the enhanced permeability and retention (EPR) effect. However, a reduction in bubble size reduces bubble acoustic response under clinical US. Microbubbles, due to their larger volume and higher acoustic response, remain for now more suitable than nano‐size UCAs. Although challenging, acoustic properties of nano‐size UCAs can be improved by shell modification and bubble aggregation strategies.^78^ Some recent examples are given bellow.

#### 2.4.2 Nanobubbles, alternative to microbubbles?

Nano‐size UCAs are nanobubbles small enough (diameter typically ranging from 100 to 600 nm) to pass through the tumor vessels into the interstitial tissues. They are composed of a phospholipid shell and a solid, liquid or gas core. Common synthetic nano‐size UCAs include gas‐filled nanobubbles (NBs), phase‐change droplets (PCDs), and echogenic liposomes (ELIPs). A wide range of other contrast agents have been reported with organic^78^ or inorganic shells.^79^ Only one article has been published on the utilization of nano‐size UCAs in pancreatic cancer imaging. It consists of an oxygen loaded NBs investigated in therapy to reduce tumor hypoxia in a mouse xenograft tumor model of human pancreatic cancer.^80^ Diagnostic imaging using nanobubble technologies have been used for other cancers such as breast,^81^, ^82^ ovarian^83^ and prostate cancer imaging.^84^, ^85^ Jiang et al. exploited NB‐Herceptin conjugates (613.0 ± 25.4 nm) for HER2‐overexpressing breast cancer imaging.^81^ In vivo, they detected a significant ultrasound contrast enhancement with targeted‐NBs compared to nontargeted‐NBs, and a prolonged retention of targeted‐NBs in HER2‐overexpressing tumors. Later, Gao and co‐workers reported on a NB that specifically targets CA‐125.^83^ The contrast agent consists of a NB covalently bound to a CA‐125 antibody (Ab‐NB), with a size as small as 74.6 ± 16.7 nm. In vivo imaging showed that at 2 min after injection the ultrasound signal was five‐fold higher for Ab‐NBs compared to nontargeted NBs suggesting that Ab‐NBs strongly accumulate in tumor and produced long‐lasting signal enhancement. Recently, Perera et al. published preclinical results using a NB targeting prostate specific membrane antigen (PSMA).^84^ PSMA‐NB (277 ± 11 nm) enabled specific tumor uptake by PSMA‐expressing tumors and selective retention, thus significantly extending duration of ultrasound signal enhancement compared to nontargeted NBs (approximately two‐fold longer). Although, these NB agents are recent, they hold promise for future opportunities for pancreatic cancer detection by ultrasound imaging. Among nano‐sized biomaterials, gas vesicles (GV) were recently developed as engineerable acoustic biomolecules for noninvasive imaging.^86^ GVs are nanostructures composed of an air‐filled protein shell (2 nm‐thick protein shell) that in nature are used by photosynthetic bacteria to regulate their flotation. Transferring the genes for making gas vesicles from the water‐dwelling bacteria into *Escherichia coli*, Shapiro and colleagues have demonstrated that the GVs can be imaged with ultrasound in the guts of mice with robust contrast enhancement.

## 3 TARGETING STRATEGIES FOR MOLECULARLY TARGETED CONTRAST AGENTS IN PDAC IMAGING

In addition to the size, the targeting moieties are also critical components of UCAs. These targeted imaging agents are designed to bind cell surface proteins upregulated in pathological tissues. Strategies for targeting ligand selection and their conjugation to contrast agents are defining steps toward development of disease‐specific UCAs.

### 3.1 Targeting ligands

**FIGURE 3:**
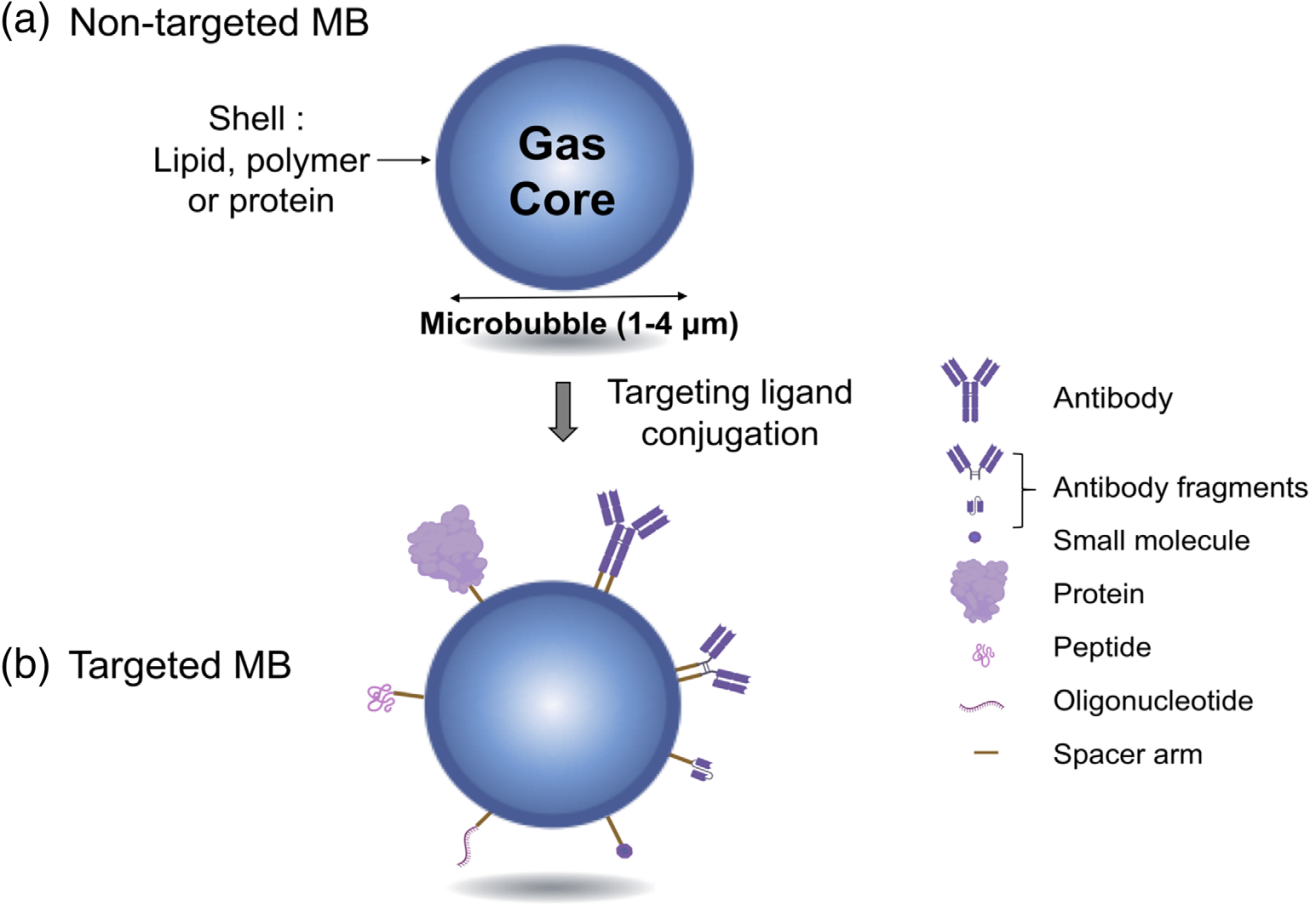
Molecularly‐targeted microbubble strategies. (a), Native microbubble consists in a gas core and a shell (lipids, polymers or proteins) and (b), can be converted into molecularly‐targeted microbubble by functionalization with various targeting ligands (antibody, fragment of antibody, protein, peptide, oligonucleotide, small molecule)

Ultrasound molecular imaging aims to selectively detect and enhance echo signals after specific binding of targeted‐contrast agents to their biological targets. Many targeting ligands have been reported in PDAC molecular imaging research (Table 3). These consist in full‐length antibodies, antibody fragments, oligonucleotides, peptides, proteins, and small molecule ligands. Such ligands may be applied for designing targeted microbubbles (Figure 3). We discuss here the various targeting ligands explored for PDAC.

**TABLE 3:** Overview of PDAC molecular imaging in preclinical studies and clinical trials

Imaging modality	Molecular target	Probe name	Targeting ligand(s)	Contrast agent	Conjugation chemistry	Animal model(s)	Results	Reference
CEUS	Thyl	MB_Thy1‐scFv_	scFv	Microbubble: phospholipid shell (DPPC, DPPE, DPPE‐PEG(5000)‐MPEG and DSPE‐PEG(5000)‐MA); gas:octafluoropropane 2 ± 0.4 μm	Thiol‐maleimide	Transgenic pancreatic cancer mouse model (Pdx1‐Cre^tg/+^; KRas^LSL/G12D/+^; Ink4a/Arf^‐/‐^)	Signal intensity MB_Thy1‐scFv_/MB_nontargeted_ in PDAC ≈ 4.4 (4 min p.i.) MB_Thy1‐scFv_ signal intensity PDAC/control pancreas ≈ 3.5 (4 min p.i.)	[16]
						Pancreatitis mouse model (L‐arginine induced)		
CEUS	VEGFR2	BR55	Heterodimer peptide	Microbubble: phospholipid shell (DSPE‐PEG(2000)); gas: perfluorocarbon, nitrogen 1.5 ± 0.1 μm	NHS	Transgenic pancreatic cancer mouse model (Pdx1‐Cre^tg/+^; KRas^LSL/G12D/+^; Ink4a/Arf^‐/‐^)	Signal intensity BR55/MB_nontargeted_ in PDAC = 3.2 (4 min p.i.) BR55 signal intensity PDAC/control pancreas ≈ 26.8 (4 min p.i.)	[73]
CEUS	CD105	Av‐PESDA‐MB	Antibody	Microbubble: Dextrose, albumin; gas: Perfluoropropane 1.1 ± 0.1 μm	Avidin‐biotin	Orthotopic human pancreatic tumor xenograft mouse model (MiaPaca‐2)	Signal intensity MB_CD101_/MB_nontargeted_ ≈ 1.4 (5 min p.i.)	[74]
						Subcutaneous pancreatic cancer mouse model (MiaPaca‐2)		
Multiphoton microscopy	Plectin‐1 receptor	Targeted bubble	Peptide (KTLLPTP)	Microbubble: phospholipid shell (DDPE‐PEG(2000); DPPC); gas: perfluorobutane; Fluorescent label: DiI 1‐5 μm	Amide coupling	In vitro study on human pancreatic cancer PANC1 cells	Targeted bubble index PANC1 /control cells ≈ 110 (20 min p.i.) bubble index MB_Targrted_/MB_nontargeted_ in PANC1 ≈ 6.1 (20 min p.i.)	[87]
MRI	Survivin	Sur‐MNP	Antisense oligonucleotide(ASON)	Nanoparticle: Chitosan core size: 12 nm; hydrodynamic size: 120 nm	NHS	Orthotopic human pancreatic tumor xenograft mouse model (BxPC‐3‐GFP cells)	T2 ratio Sur‐MNP/MNP_nontargeted_ in PDAC ≈ 0.76 (40 hr p.i) T2 ratio Sur‐MNP/MNP_nonsense_ in PDAC ≈ 0.80 (40 hr p.i.)	[88]
MRI	Neuropilin‐1	4‐Nanocage	Peptide (iRGD)	Nanoparticle: Gd‐DTPA‐mal, Hsp 16. 37.1 nm	Thiol‐maleimide	Transgenic pancreatic cancer mouse model (Kras^G12D^; Trp53^R172H^; Pdx‐1Cre)	4‐nanocage tumor‐to‐normal tissue contrast ratio ≈ 1.23 (60 min p.i.) Nanocage_nontargeted_ tumor‐to‐normal tissue contrast ratio ≈ 1.03 (60 min p.i.)	[89]
MRI	IGF1 receptor	IGF1‐IONP‐dox	Peptide (IGF1)	Nanoparticle: IONPs with doxorubicin 14.5‐20.4 nm	NHS	Orthotopic human pancreatic tumor xenograft mouse model (surgically resected human pancreatic cancer tissue)	Signal intensity IGF1‐IONP/IONP_nontargeted_ in PDAC ≈ 0.6 (24 hr p.i.) tumor weight ratio IGF1‐IONP‐Dox/IONP‐Dox ≈ 0.6 tumor weight ratio IGF1‐IONP‐Dox/Dox ≈ 0.5	[90]
MRI	MUC4, CEACAM6, CD44v6	IONPs‐PEG‐MCC triple scAbs	scAbMUC4, scAbCEACAM6, scFvCD44v6	Nanoparticle: IONPs 23.6 nm	NHS	Subcutaneous pancreatic cancer mouse model (BxPC‐3 cells)	T2 ratio IONP_mono‐targeted_ 6 hr p.i./pre.i in PDAC ≈ 0.68 T2 ratio IONP_bi‐targeted_ 6 hr p.i./pre.i in PDAC ≈ 0.53 T2 ratio IONPs‐PEG‐MCC scAbs 6 hr p.i./pre.i in PDAC ≈ 0.4 tumor weight ratio IONPs‐PEG‐MCC scAbs/IONPs‐PEG ≈ 0.37 (18 days p.t.)	[91]
MRI	MUC‐1	MUC1‐SPION	Antibody	Nanoparticle: SPIONs 63.5 ± 3.2 nm	Amide coupling	Subcutaneous pancreatic cancer mouse model (BxPC‐3 cells)	Apparent decrease of signal tumo with MUC1‐SPION compared to control (4 hr p.i.)	[92]
MRI	Folate receptor	SPIO‐PEG(2000)‐FA	Folic acid	SPION with PEG(2000) 32 nm	Amide coupling	Subcutaneous pancreatic cancer mouse model (KB cells)	Tumor signal intensity SPIO‐PEG(2000)‐FA 2 hr p.i/preinjection≈ 0.8	[93]
MRI	Folate receptor	PEG‐FA‐ICGDER02‐chi	Folic acid	Chitosan‐gemcitabine nanoparticle 200–300 nm	Amide coupling	Orthotopic human pancreatic tumor xenograft mouse model (COLO357)	Tumor signal intensity PEG‐FA‐ICGDER02‐chi/NP_nontargeted_≈ 2 (24 hr p.i.)	[94]
MRI ‐ FI	Plectin‐1	Plectin‐SPION‐Cy7	Antibody	Nanoparticle: SPION and cyanine 7 core size: 12 nm, hydrodynamic size: 84 nm	NHS	Orthotopic human pancreatic tumor xenograft mouse model (XPA‐1‐RFP cells)	T2 ratio Plectin‐SPION‐Cy7 48 hr p.i./pre.i in PDAC ≈ 0.8 T2 ratio Plectin‐SPION‐CyT/SPION‐Cy7_nontargeted_ in PDAC ≈ 0.8 (48 hr p.i.)	[95]
MRI ‐ FI	uPAR	(DGL)‐U11	Peptide (U11)	Nanoparticle: Dendron‐grafted polylysine (DGL)‐Gd and fluorescent cyanine dye Cy5.5 22.98 nm	NHS	Pancreatic tumor progression rat model (DMBA)	FI signal intensity increase with disease severity (24 hr p.i.) weak FI signal with DGL_nontargeted_ compared to (DGL)‐U11 (24 hr p.i.) no MRI signal difference between PanIN‐1 and PanIN‐II(24 hr p.i.) MRI signal in the tumor appears at PanIN‐III (24 hr p.i.) apparent increase of MRI signal in PDAC compared to PanIN‐III (24 hr p.i.)	[96]
MRI ‐ FI	Glypican‐1	Gd‐au‐NC‐GPC‐1	Antibody	Nanoparticle: Gold nanochister, Gd 13.5–24.4 nm	NHS	Subcutaneous pancreatic cancer mouse model (COLO‐357)	FI intensity Gd‐Au‐NC‐GPC‐1/Gd‐Au_nontargeted_ in PDAC ≈ 1.2 (30 min p.i.) MRI intensity Gd‐Au‐NC‐GPC‐1/Gd‐Au_nontargeted_ in PDAC ≈ 1.7 (30 min p.i.)	[97]
MRI ‐ FI	Glypican‐1	ORI‐GPC1‐NP	Antibody	Nanoparticle: Gold nanocages, Cy7, Gd with oridonin 88 nm	NHS	Orthotopic human pancreatic tumor xenograft mouse model (BXPC‐3‐GFP)	FI signal intensity ORI‐GPC1‐NP/ORI‐NP in PDAC ≈ 2.5 (24 hr p.i.) MRI signal intensity ORI‐GPC1‐NP/ORI‐NP in PDAC ≈ 2.1 (24 hr p.i.) tumor weight ratio ORI‐GPC1‐NP/ORI‐NP ≈ 0.27 tumor weight ratio ORI‐GPC1‐NP/ORI ≈ 0.36 (after 14 days) tumor weight ratio ORI‐GPC1‐NP/GEM ≈ 0.5 (after 14 days)	[98]
MRI ‐ FI	Glypican‐1	GPC1‐GEM‐NP	Antibody	Nanoparticle: Gold nanocages, Cy7, Gd with gemcitabine 60 nm	NHS	Orthotopic human pancreatic tumor xenograft mouse model (BXPC‐3‐GFP)	FI signal intensity GPC1‐GEM‐NP/GEM‐NP in PDAC ≈ 3.5 (24 hr p.i.) MRI signal intensity GPC1‐GEM‐NP/GEM‐NP in PDAC ≈ 2.5 (24 hr p.i.) tumor weight ratio GPC1‐GEM‐NP/GEM‐NP ≈ 0.25 (after 14 days) tumor weight ratio GPC1‐GEM‐NP/GEM ≈ 0.6 (after 14 days)	[99]
MRI ‐ FI	CD326	UPG‐CD326	Antibody	Nanoparticle: Gadolinium ion‐doped upconversion nanoparticles (UCNPs), DSPE, DSPE‐PEG(2000)‐Mal	Thiol‐maleimide	Subcutaneous pancreatic cancer mouse model (BxPc‐3)	FI signal intensity UPG‐CD326/UPG in PDAC ≈ 4 (24 hr p.i.) MRI signal intensity UPG‐CD326/UPG in PDAC ≈ 1.4 (24 hr p.i.)	[100]
MRI ‐FI	Plectin‐1 & integrin β4	Gd‐Cy7‐PTP/RGD	Peptides (PTP (short for KTLLPTP) & RGD)	Nanoparticle: Gd‐DOTA, Cy7	NHS	Orthotopic human pancreatic tumor xenograft mouse model (Panc1)	FI signal intensity Gd‐Cy7‐PTP/RGD/free Cy7 in PDAC ≈ 2 (4 hr p.i.) MRI Gd uptake Gd‐Cy7‐PTP/RGD/free Gd in PDAC ≈ 1.6 (4 hr p.i.)	[101]
CEST MRI	uPA	GR‐ 4Am‐SA	Peptide (RG)	4‐aminosalicylic acid	Amide coupling	Subcutaneous pancreatic cancer mouse model (Capan‐2)	GR‐4Am‐SA signal (9.5 ppm) in PDAC = 10.6% ± 2.8% GR‐4Am‐SA signal (5 ppm) in PDAC = 2.2% ± 1%	[102]
CEST MRI	Extradomain‐B fibronectin	Dex10‐ZD2	Peptide (ZD2)	Dextran 8.2 ± 1.73 nm	NHS	Subcutaneous pancreatic cancer mouse model (KPC)	Signal Dex10‐ZD2/Dex10 in PDAC ≈ 11.7 (45 min p.i.)	[103]
FI	Integrin αvβ6	R01‐MG‐IRDye800	Peptide (R01‐MG)	IRDye800	NHS	Subcutaneous pancreatic cancer mouse model (BxPC‐3‐luc2)	R01‐MG‐IRDye800 tumor to background ratio ≈ 2.5 (5 hr p.i.) tumor to background ratio R01‐MG‐IRDye800/IRDye800 ≈ 2.5 (24 hr p.i.) R01‐MG‐IRDye800 tumor to background ratio tumor/control ≈ 3 (24 hr p.i.)	[104]
						Orthotopic human pancreatic tumor xenograft mouse model (AsPC1)		
						Transgenic pancreatic cancer mouse model (Pdx1‐Cre^tg/+^; KRas^LSL G12D/+^; Ink4a/Arf^‐/‐^)		
FI	PEPT1	NPs‐DIP	Peptide (Ser–Glu = DIP)	Nanoparticle, NIR775 dye 59.1 nm	NHS	Subcutaneous pancreatic cancer mouse model (AsPC‐1)	NPs‐DIP tumor‐to‐background ratio ≈ 5 (20 hr pi) nontargeted NPs tumor‐to‐background ratio ≈ 1.4 (20 hr pi)	[105]
FI	MMP14	Cy5‐M17	DNA aptamer (M17)	Cy5	Streptavidin‐Biotin	Subcutaneous pancreatic cancer mouse model (MIA PaCa‐2)	Apparent signal in tumor with Cy5‐M17 (3 min p.i.) no apparent signal in tumor with Cy5‐labeled library (1 hr p.i.)	[106]
FI	MMPs	I_780_BP‐PEG12	Peptide	Fluorophore IR780 and BHQ‐3 quencher	Michael‐type addition	Orthotopic human pancreatic tumor xenograft mouse model (Panco2)	I780BP‐PEG12 signal intensity PDAC/control pancreas ≈ 8.7 (6 hr p.i.)	[107]
FI	PL45 cells	Cy5‐XQ‐2d	DNA aptamer (XQ‐2d)	Cy5	Annealing by Primer sequence	Subcutaneous pancreatic cancer mouse model PL4(5 cells)	Apparent signal in tumor with Cy5‐XQ‐2d (1 hr p.i.) no apparent signal in tumor with Cy5‐labeled library (1 hr p.i.)	[108]
FI	CEA	SGM‐101	Antibody	Fluorophore BM104	NHS	12 PDAC patients with CEA levels >3 ng/ml	Tumor‐to‐background ratio = 1.6 ± 0.37 (primary tumors) rumor‐to‐background ratio—1.7 ± 0.42 (metastases)	[109]
FI	Mucin1	Anti‐hMUC1 ab ‐ DyLight 755	Antibody	DyLight 755	NHS	Subcutaneous pancreatic cancer mouse model (Capan‐2)	Apparent signal in PDAC with Anti‐hMUC1 Ab—DyLight 755 (24 hr p.i.) no signal in PDAC with normal IgG—DyLight 755N	[110]
FI	Tissue factor	1849‐ICG	Antibody	ICG	NHS	Subcutaneous pancreatic cancer mouse model (BxPC‐3)	Signal intensity 1849‐ICG in PDAC ≈ 16 (24 hr p.i.) Survival 1849‐ICG PIT/ 1849‐ICG ≈ 2.5 (after 24 days)	[111]
FI	Integrin αvβ6	Dye‐SA‐B‐HK	Peptides	IRDye700	Streptavidin‐Biotin	Subcutaneous pancreatic cancer mouse model (BxPC‐3)	Signal intensity dye‐SA‐B‐HK/dye‐SA‐B ≈ 1.9 (2 hr p.i.) tumor volume dye‐SA‐B‐HK PT/dye‐SA‐B‐HK ≈ 0.4 (34 days) tumor volume dye‐SA‐B‐HK PT/dye‐SA‐B PT ≈ 0.5 (34 days)	[112]
FI	Folate receptor	bMSN@Cy7.5‐FA NP	Folic acid	MSN, Cy7.5 102.6 ± 0.2 nm	NHS	Subcutaneous pancreatic cancer mouse model (BxPC‐3)	Tumor uptake bMSN@Cy7.5‐FA NP/NP_nontargeted_ ≈ 1.6 (12 hr p.i.)	[113]
FI ‐ PAI	EGFR	Cetuximab‐IRDye800	Antibody	IRDye800	NHS	Seven patients with resectable pancreatic masses suspected to be PDAC	Antibidy‐IRDye800 fluorescence signal intensity PDAC/control tissue ≈ 4.5 photoacoustic tumor to background ratio in PDAC ≈ 3.7 sensitivity = 96.1 % specificity = 67.0%	[114]
PET	Integrin αvβ6	^64^Cu DOTA‐R_0_1‐MG	Peptides (R_0_1‐MG & R_0_1‐MG‐F2)	^64^Cu or ^18^F or ^68^Ga	NHS	Subcutaneous pancreatic cancer mouse model (BxPC‐3)	Mice: ^18^FFP‐R_0_1‐MG‐F2 tumor‐to‐muscle ratio PDAC/control pancreas ≈ 5.6 (1 hr p.i.) ^64^Cu DOTA‐R_0_1‐MG tumor‐to‐muscle ratio PDAC/control pancreas ≈ 6 (1 hr p.i) ^68^GaNODAGA‐R_0_1‐MG tumor‐to‐muscle ratio PDAC/control ≈ 5 (1 hr p.i.)	[47]
		^68^GaNODAGA ‐R_0_1‐MG				5 healthy patients and 2 PDAC patients		
		^18^FFP‐R_0_1‐MG‐F2				Clinic: ^18^FFP‐R_0_1‐MG‐F2 SUVmean ratio PDAC/control pancreas ≈ 3.1 (1 hr p.i.) ^68^GaNODAGA‐R_0_1‐MG SUVmean ratio PDAC/control pancreas ≈ 2.3 (1 hr p.i.)		
PET	EGFR & HER3	^89^Zr‐MEHD7945A	Antibody (MEHD7945A)	^89^Zi	NHS	Subcutaneous pancreatic cancer mouse model (BxPC‐3 & AsPC‐1)	AsPC‐1 tumor uptake ^89^Zr‐MEHD7945A ≈ 6.2 (72 hr p.i.) AsPC‐1 tumor uptake ^89^Zr‐IgG ≈ 1.0(72 hr p.i.) BxPC‐3 tumor uptake ^89^Zr‐MEHD7945A ≈ 9 (72 hr p.i.) BxPC‐3 tumor uptake ^89^Zr‐IgG ≈ 0.6 (72 hr p.i.)	[115]
PET	EGFR	LPEI‐PEG‐GE11‐NIS	Peptide (GE11 = CYHWYGYTPQNVI)	^131^I	NHS	Transgenic pancreatic cancer mouse models (Ptf1^a+/Cre^; Kras^+/LSL‐G12D^; Trp53^loxP/loxP^, and Ptf1^a+/Cre^; Kras^+/LSL‐G12D^; Trp53^loxP/loxP^; Egfr^/f1/f1^)	Tumor uptake LPEI‐PEG‐GE11‐NIS ≈ 14.2 (48 hr p.i.) tumor uptake LPEI‐PEG‐GE11‐antisenseNIS ≈ not significant (48 hr p.i.) median survival LPEI‐PEG‐GE11‐NIS ≈ 25 days (after 1 week treatment) median survival LPEI‐PEG‐GE11‐antisenseNIS ≈ 11 days (after 1 week treatment)	[116]
PET	Extradomain B fibronectin	ZD2‐^68^Ga‐NOTA	Peptide (ZD2 = TVRTSAD)	^68^Ga	Amide coupling	Subcutaneous pancreatic cancer mouse model (BxPC3 & Capan‐1)	ZD2‐(^68^Ga‐NOTA) tumor‐to‐muscle ratio BxPC‐3 ≈ 6 (1 hr p.i.) ZD2‐(^68^Ga‐NOTA) tumor‐to‐muscle ratio Capan‐1 ≈ 6.4 (1 hr p.i.)	[117]
PET	EIIIB fibronectin	^64^Cu‐NJB2	Nanobody (NJB2)	^64^Cu	Sortase‐mediated transacylation	Subcutaneous pancreatic cancer mouse model (KPC)	PanINs pancreas‐to‐muscle ratio ^64^Cu‐NJB2/ ^18^F‐FDG ≈ 4.5 (2 hr p.i.) PDAC pancreas‐to‐muscle ratio ^64^Cu‐NJB2/^18^F‐FDG ≈ 1.3 (2 hr p.i.)	[118]
PET	Integrin αvβ6	^68^Ga‐cycratide	Peptide (cyclo‐RGDLATLK‐)	^68^Ga	NHS	Subcutaneous pancreatic cancer mouse model (BxPC‐3)	Mice: Tumor uptake ^68^Ga‐cycratide/^68^Ga‐linear‐pep ≈ 2.7 (30 min p.i.)	[48]
						Orthotopic human pancreatic tumor xenograft mouse model (BxPC‐3)		
							Five healthy volunteers and two patients PDAC suspected	Clinic: Average ^68^Ga‐cycratide SUVmax ≈ 3.2 average ^18^F‐FDG SUVmax ≈ 4.5
PET	Tissue factor & CD105	^64^Cu‐labeled heterodimer	Heterodimer fab	^64^Cu	Isothiocyanate–amine coupling	Subcutaneous pancreatic cancer mouse model (BxPC‐3)	Tumor uptake ^64^Cu‐labeled heterodimer/^64^Cu‐labeled monomer(s) ≈ 3 (30 hr p.i.) ^64^Cu‐labeled heterodimer signal intensity PDAC/control pancreas ≈ 17.1 (30 hr p.i.)	[119]
						Orthotopic human pancreatic tumor xenograft mouse model (BxPc‐3)		
		^64^Cu‐NOTA‐ALT‐836‐fab						
		^64^Cu‐NOTA‐TRC105‐fab						
PET	MT1‐MMP	^68^Ga‐DOTA‐AF7p	Peptide (MT1‐AF7p) = HWKHLHNTKTFL)	^68^Ga or ^89^Zr	Isothiocyanate–amine coupling	Orthotopic human pancreatic tumor xenograft mouse model (CAPAN‐2)	Average tumor uptake ^89^Zr‐DFO‐LEM2/15/^68^Ga‐DOTA‐AF7p ≈ 27 (90 min p.i.)	[120]
		^89^Zr‐DFO‐LEM2/15	Antibody (LEM2/15)			Subcutaneous pancreatic cancer mouse model (CAPAN‐2)		
PET	Integrin αvβ6	^64^Cu‐DOTA‐peptide	Peptides R_0_2, E_0_2, S_0_2 and R_o_1 (derived motif RTDLXXL)	^64^Cu	NHS	Orthotopic human pancreatic tumor xenograft mouse model (BxPC‐3)	Tumor‐to‐muscle ratio ^64^Cu‐DOTA‐peptides ≈ 9.5 (1 hr p.i.)	[121]
						Subcutaneous pancreatic cancer mouse model (BxPC‐3)		
PET/CT ‐ FI	CA19‐9	^ss^DFO‐5B1	Antibody (5B1)	^89^Zr‐Desferrioxamine (DFO) or NIRF dye (FL)	Azide cycloaddition	Subcutaneous pancreatic cancer mouse model (BxPC3)	Tumor uptake ^ss^dual‐5B1 ≈ 103 (120 hr p.i.) tumor uptake ^ss^DFO‐5B1 ≈ 114.1 (120 hr p.i.)	[122]
		^ss^FL‐5B1				Orthotopic human pancreatic tumor xenograft mouse model (Suit‐2)		
		^ss^dual‐5B1						
PET/CT	CA19‐9	MVT‐2163	Antibody (HuMab‐5B1)	^89^Zr	Isothiocyanate–amine coupling	12 patients with CA19‐9–positive metastatic malignancies	Average MVT‐2163 SUV = 101.4 (7 days p.i) average MVT‐2163 lesion‐to‐blood ratio = 18.4 (7 days p.i)	[123]
SPECT	Claudin‐4	^111^Inanti‐claudin‐4 mAb	Antibody (MAB4219)	^111^In	Isothiocyanate–amine coupling	Subcutaneous pancreatic cancer mouse model (Panc‐1)	Tumor uptake ^111^Inanti‐claudin‐4 mAb/^111^InmIgG ≈ 2.5 (72 hr p.i.)	[124]
						Transgenic pancreatic cancer mouse model (Kras^G12D^; Trp53^R172H^; Pdx‐1Cre)		
SPECT	Tissue factor	^111^In‐1849	Antibody	^111^In	Isothiocyanate–amine coupling	Subcutaneous pancreatic cancer mouse model (BxPC‐3)	^111^In‐1849 signal intensity in tumor ≈ 50.6 (4 days p.i.) ^111^In‐1849 signal intensity in muscle ≈ 0.91 (4 days p.i.) ^111^In‐1849 signal intensity in blood ≈ 10.1 (4 days p.i.)	[125]
MSOT	pH‐uPAR	MSN‐UPA	Chitosan and uPA	MSNs 115 ± 10 nm	NHS	Orthotopic human pancreatic tumor xenograft mouse models (S2VP10)	Tumor signal intensity MSN‐UPA ≈ 47 (4 hr p.i.)	[126]
MSOT ‐ FI	EGFR	EGF‐750 probe	Peptide	CF‐750 dye	NHS	Orthotopic human pancreatic tumor xenograft mouse model (S2VP10L)	Tumor signal intensity EGF‐750 probe ≈ 318 (6 hr p.i.) tumor signal intensity EGF‐750 ≈ 0 (6 hr p.i.)	[127]
MSOT ‐ FI	Integrin αvβ3	Syndecan‐1 probe	Syndecan‐1	ICG	NHS	Orthotopic human pancreatic tumor xenograft mouse model (S2VP10L)	Tumor signal intensity Syndecan‐1 probe ≈ 480 (6 hr p.i.) tumor signal intensity ICG control dye ≈ 0.0003 (6 hr p.i.)	[128]

Abbreviations: ASON, antisense oligonucleotide; CD, cluster of differentiation; CEA, carcinoembryonic antigen; CEACAM6, CEA Cell Adhesion Molecule 6; Cy, cyanine dye; DGL, dendron‐grafted polylysine; DPPC, 1,2‐dipalmitoylphosphatidylcholine; DPPE, 1,2‐Dipalmitoyl‐snglycero‐3‐phosphoethanolamine; DPPE‐PEG(5000)‐MPEG, 1,2‐Dipalmitoyl‐sn‐glycero‐3‐phosphoethanolamine‐*N*‐ carbonyl‐methoxypolyethyleneglycol(polyethylene glycol)‐5000; DSPE, 1,2‐distearoyl‐sn‐glycero‐3‐phosphoethanolamine; DSPE‐PEG(2000), 1,2‐distearoyl‐sn‐glycero‐3‐phosphoethanolamine‐*N*‐amino(polyethylene glycol)‐2000; DTPA‐mal, diethylenetriamine‐pentaacetic‐maleimide; EGFR, epidermal growth factor receptor; Gd, gadolinium; GEM, gemcitabine; HER3, receptor tyrosine‐protein kinase erbB‐3; Hsp, heat shock protein; ICG, indocyanine green; IONP, magnetic iron oxide nanoparticle; MMP, matrix metalloproteinase; MSN, mesoporous silica nanoparticle; MSOT, optoacoustic tomography; MUC1, mucin‐1; MUC4, mucin‐4; ND, not determined; NHS, *N*‐hydroxysuccinimide; NP, nanopaticle; ORI, oridonin; PanIN, pancreatic intraepithelial neoplasia; PEPT1, human peptide transporter 1; SPION, superparamagnetic iron oxide nanoparticle; SUV, standardized uptake value; UCNP, gadolinium ion‐doped upconversion nanoparticle; uPA, urokinase‐type plasminogen activator; uPAR, urokinase‐type plasminogen activator receptor.

#### 3.1.1 Full‐length antibodies

Among targeting ligands, antibodies (molecular weight ~150 kDa) have been the most widely adopted for active targeting in cancer imaging.^129^ Several preclinical and clinical imaging studies have exploited monoclonal antibodies as targeting ligands.^92^, ^95^, ^97^, ^98^, ^99^, ^100^, ^109^, ^110^, ^111^, ^114^, ^115^, ^122^, ^123^, ^124^, ^125^, ^130^ There are many advantages in the use of antibodies as molecular imaging probes. First, a wide range of humanized antibodies targeting endothelial molecules are commercially available. Antibodies are bivalent and have the inherent ability to bind to antigens with high affinity and high specificity. However, their utilization is limited due to time‐consuming and expensive development processes.^131^ Importantly, their long blood circulation times (ranging from days to weeks) and slow background clearance rate (optimal tumor uptake between 2‐ and 5‐days postinjection) further limits the application of full‐length antibodies as ideal imaging agents for clinical practice. Considering the relatively short half‐life of most microbubbles (<10 min), it is important to select ligands with compatible clearance time to minimize unnecessary side‐effects and to achieve high imaging signal in a relatively shorter timeframe. Therefore, lower molecular weight ligands are being preferred for the development of targeted UCAs.

#### 3.1.2 Antibody fragments

Imaging probes using antibody fragments (e.g., Fab or Fab′ (50/55 kDa), scFv (26 kDa) and F(ab′)_2_ (110 kDa)) or a combination of antibody fragments (e.g., diabodies (55 kDa), minibodies (75 kDa), triabodies (90 kDa), and tetrabodies (120 kDa)) are being frequently used.^132^ It has been recognized that blood clearance rate is inversely related to the size of the protein that is, clearance rate of scFV > Fab or Fab′ > diabody > minibody > triabody > F(ab′)_2_ > tetrabody > IgG.^133^

For PDAC ultrasound imaging with targeted microbubbles, Bam et al. used a scFv ligand identified and engineered from a yeast‐surface‐display technique panned against human Thy1 protein.^72^ Zou and co‐workers expoited a trio of two single chain antibodies and one scFv conjugated to magnetic iron nanoparticles targeted three overexpressed proteins in PDAC (MUC4, CEACAM6, and CD44v6, respectively).^91^ They successfully showed the advantage of the triple construct over the single ones with a significant T2 value change by preclinical MRI. Interestingly, multiple target protein imagings were published by Luo et al. using a heterodimer of Fab for PET molecular imaging targeting both tissue factor and CD105.^119^ In comparison to full‐length antibodies, the use of scFv has shown to be advantageous due to a minimized immunogenicity and good safety in preclinical animal studies. Moreover, they exhibit good stability and solubility with rapid blood clearance, and are a viable means of reducing off‐target effects. Furthermore, scFvs are versatile and can be easily modified by adding site‐specific tags for detection and bioconjugation. They are cost effective and easier for scale up production. The major limitation of scFvs is their monovalency (one antigen binding site), which could result in modest retention time and potentially decreased imaging window. To prolong their mean retention time, molecules can be conjugated to polyethylene glycol (PEG) polymers.^134^ On the other hand, numerous approaches to genetically engineer multivalent fragments have been pursued.^135^ Using scFvs as building blocks, fragments such as scFv polymers and scFv‐fusion proteins have been generated. As examples, diabodies and minibodies form stable molecules that have been shown to have a longer blood clearance and improved tumor uptake due to their larger size and multivalence properties.^136^

As the smallest protein scaffolds, the preclinical imaging use of nanobodies (12 kDa) and affibodies (7 kDa) have become interesting alternatives to scFvs. A recent study reported a high‐affinity nanobody, named NJB2, specific for the alternatively spliced domain EIIIB of fibronectin overexpressed in ECM diseases and neovasculature.^118^ Jailkhani et al., identified that EIIIB is expressed in the periphery of pancreatic intraepithelial neoplasia (PanIN), a microscopic lesion of the pancreas and precursor of PDAC, and that its expression increases with PDAC progression in mouse models and human patient samples. On the other hand, the rapid blood clearance of affibodies and their high tumor penetration generally provide good contrast shortly after injection. A recently engineered affibody conjugated to commercial microbubbles (MB_ABY‐B7‐H3_) targeted against B7‐H3, a T‐cell modulator, was applied preclinically in breast cancer models for ultrasound molecular imaging.^137^ MB_ABY‐B7‐H3_ enhanced B7‐H3 molecular signal in breast tumor models, improving cancer early detection, while offering the advantages of a small size ligand and easier production for potential clinical imaging. B7‐H3 is also overexpressed by the tumor‐associated neovasculature in pancreatic cancer and MB_ABY‐B7‐H3_ or nanobubbles functionalized with B7‐H3‐specific affibodies could prove useful for PDAC detection by ultrasound molecular imaging.^138^

As an alternative to antibodies, several other classes of ligands are currently employed for targeting pancreatic cancer. Examples below include peptides, proteins, oligonucleotides and small molecules.

#### 3.1.3 Oligonucleotides

Aptamers are a class of short single‐stranded oligonucleotides (DNA or RNA) that can bind to cellular targets with high affinity and specificity, and can inhibit protein function. When chemically modified, aptamers show enhanced stability, tissue permeability, and lower immunogenicity compared to antibodies.^139^ Wu et al., presented a DNA aptamer with high affinity to the PDAC tumors of PL45 cell line.^108^ In clinical samples, the recognition ability of the aptamer by fluorescence for PDAC tissues was 82.5%, but with control false positive rate of 25%. An important point to consider is that aptamers are sensitive to their environment, particularly they are cleavable by nucleases, and thus often necessitate synthesis of oligonucleotides using chemically modified nucleotides to enhance their half‐life in nuclease rich blood circulation.^140^ Recently, Huang et al., published a DNA aptamer targeting the matrix metalloproteinase 14 (MMP14), and described its potential for in vivo diagnosis of MMP14‐positive cancer by fluorescence.^106^ Additionally, Wang and co‐workers published an interesting in vivo study using antisense oligonucleotides for survivin imaging by decreasing the T2 value on MRI.^88^ To the best of our knowledge, no microbubble modified with oligonucleotides has been published for pancreatic cancer detection. Development of microbubbles conjugated with aptamers, for example, would allow a highly specific target protein binding without imaging penetration depth limitation and can have better clinical translation possibilities.

#### 3.1.4 Peptides

Small peptides, usually less than 50 amino acids, are advantageous over other classes of targeting ligands due to their good tissue and cell penetration, easy production with lower cost, compared to the expensive production of monoclonal antibodies in hybridoma cell cultures.^141^ Peptide ligands have high potential for clinical applications in a wide range of pathologies.^142^

Microbubbles have been reported to be capable of bearing a variety of peptide ligands targeted to endothelial cell adhesion molecules (i.e., P‐selectin, vascular cell adhesion molecule‐1 [VCAM‐1]), von Willebrand factor (VWF), and oxidized low‐density lipoprotein cholesterol receptor‐1 (LOX‐1)) for imaging the recruitment of inflammatory cells and/or platelets.^143^ In the context of pancreatic cancer imaging, a wide range of biomarkers have been imaged through peptides as targeting ligands. Those targets are proteins mainly located on plasma membrane such as neuropilin‐1,^89^ EGFR,^116^, ^127^ uPAR,^96^ and integrin αvβ6,^47^, ^48^, ^104^, ^112^, ^121^ and several others have tested theranostic application of peptides in various models.^48^, ^90^, ^112^, ^116^ Moreover, the peptide transporter 1 (PEPT1) is also investigated as another relevant biomarker of pancreatic cancer. The Ser‐Glu dipeptide was used as a PEPT1 ligand,^144^ later functionalized with nanoparticles and evaluated in pancreatic cancer using FI^127^. The probe successfully accumulated in the tumor with a signal intensity about 3.6‐fold higher than in the normal tissue. Of high interest, plectin‐1 and integrin β4, have been detected through the utilization of a bispecific molecular probe and allowed imaging of pancreatic neoplasms and angiogenesis simultaneously.^101^ Such methodologies can greatly increase the targeting efficiency compared to that of either single peptide. Additionally, an in vitro proof‐of‐principle study performed for plectin‐1 receptor imaging using peptide‐labeled microbubbles using multiphoton microscopy.^87^ Such imaging modality has the ability to detect directly the microbubbles, without the need of contact medium (ultrasound gel) between the transducer and the zone being imaged. The motivation here has been to develop a technology using targeted microbubbles to ensure that all pancreatic cancerous tissues has been removed during resection procedures.

Peptides also have the capacity to target markers located intracellularly or within the extracellular matrix. This property was exploited to visualize fibronectin in pancreatic cancer environment using PET imaging.^117^ Preclinical quantitative analysis revealed that the radiolabeled heptapeptide probe upon injection results in more than five‐fold uptake in tumors as compared to the surrounding muscle tissues. With similar results, Han et al., reported the use of a high biocompatible dextran‐peptide probe for chemical exchange saturation transfer magnetic resonance imaging (CEST MRI) of PDAC.^103^ Growth and migration of cancer from a primary tumor to a site of metastasis require the ability of cells to penetrate surrounding tissues and endothelial wall to enter the blood circulation, to degrade and to remodel them. Tumor cell invasion requires the dissolution of extracellular matrix (ECM) and passage of malignant cells through the vascular basement membrane by intense enzyme activities. Of high interest, cleavable peptides allow to assess pathological proteolytic activities occurring within the tumor site. Specific cleavable peptides have been used to detect PDAC by imaging the activity of proteases including matrix metalloproteinases (MMPs),^107^, ^120^ urokinase‐type plasminogen activator (uPA)^102^ and cathepsin E.^145^ Particularly, based on an increasing in vivo fluorescence signal intensity Li and co‐workers were able to differentiate between normal pancreas, PanIN‐I, PanIN‐II/PanIN‐III grades and PDAC using cathepsin E cleavable peptide.^145^ Additionally, a study compared the in vivo performances of MT1‐MMP antibody and MT1‐MMP peptide probe for accurate PDAC detection. Both probes accumulated in MT1‐MMP‐expressing tumors, although the antibody‐based probe exhibited 25‐30‐fold higher tumor uptake than the peptide.^120^ Other researchers posted that the mutagenesis of key amino acid residues could optimize the binding ability of MT1‐MMP‐peptide ligand.^146^ In breast cancer mouse model, Ren and co‐workers enhanced the fluorescence signal in tumor by 3.2‐fold compared to the unmodified peptide.

#### 3.1.5 Proteins

Recognition and targeting of pancreatic cancer biomarkers can also be done by proteins. Those proteins have the capacity to bind receptors as illustrated by the serine protease urokinase‐type plasminogen activator (uPA) and its receptor (uPAR) used in preclinical research for PDAC imaging.^126^ Additionally, integrin αvβ3 is a key regulator of adhesion and signaling in numerous biological processes, including tumor cell migration, metastasis, and angiogenesis. Integrin αvβ3 appears to be functionally coupled with syndecan‐1 molecule that regulates its activity during carcinoma cell spreading and migration. Using a syndecan‐1 based probe, integrin αvβ3 expression was assessed by optoacoustic tomography in PDAC mouse models.^128^

#### 3.1.6 Small molecules

Small molecule ligands have an easy tumor penetration due to their smaller sizes (typically <500 molecular weight). The rapid systemic clearance and facility to synthetize and modify are other features making small molecule ligands attractive. One example of small molecule is folic acid (FA), also known as vitamin B9. FA is vital for the rapid proliferation of tumor cells and its receptor, the glycosylphosphatidylinositol‐anchored protein folate receptor, is overexpressed in various types of human tumors. In contrast, healthy cells express low levels of folate receptors. Several studies have focused on molecular imaging of pancreatic cancer through FA‐conjugated nanoparticles by MRI^93^, ^94^ and FI.^113^ Folate has also been integrated into the lipid membrane of microbubbles and tested for binding specificity to human ovarian carcinoma SKOV3 cells.^147^ Folate‐targeted microbubbles showed high affinity to SKOV3 cells with folate receptor overexpression. However, the current trend for the application of FA targeting is more favorable for the development of nanoparticles because of their capacity to extravasate vascular system.^148^, ^149^, ^150^, ^151^

### 3.2 Ligand—contrast agent conjugation strategies

Different strategies have been employed for converting nontargeted microbubbles into targeted microbubbles for ultrasound molecular imaging. These are mainly by conjugation of targeting ligands onto the microbubble surface. The coupling can be directly on the microbubble shell, but more frequently a polyethylene glycol spacer arm is added to display the ligands away from the microbubble surface.^152^

The most common conjugation strategy is the biotin‐(strept)avidin linkage.^74^, ^106^, ^112^ Each streptavidin molecule consists of four binding pockets for biotin with noncovalent interaction of dissociation constant in the femtomolar range. However, this approach is restrained to proof of concept in preclinical studies due to the potential immunogenicity associated with (strept)avidin, and the cross reactivity of unconjugated (strept)avidin to endogenous biotin in human body.^153^ Thus, targeted bubbles beyond biotin‐(strept)avidin conjugation chemistry have been developed for clinical permissibility. These approaches rely on the formation of covalent bonds between the ligand and functional groups presented by PEG spacer arms. A type of chemistry used for covalent conjugation labeling of microbubbles with ligands is amide bond formation with *N*‐hydroxysuccinimide (NHS)–ester and its derivatives. The NHS functional group on microbubble shell forms a bond with amine groups in protein ligands but is less controllable than maleimide chemistry because of the presence of multiple amine groups in peptides and proteins.^154^ Maleimide‐thiol conjugation chemistry has proven to be clinically translatable as evidenced by its adoption in many FDA approved antibody‐drug conjugates.^155^ The maleimide on the extremity of the PEG spacer arm allows covalent conjugation to a thiol group on the targeting agent, (e.g., a terminal cysteine residue). This strategy permits site‐specific conjugation of targeting molecules to microbubbles with possibly lower target‐ligand steric hindrances.

## 4 PRECLINICAL MODELS FOR PDAC AND PANCREATITIS DIFFERENTIATION BY ULTRASOUND IMAGING

The ability of imaging tools to differentiate between PDAC and pancreatitis tissue is crucial. Numerous rodent models have contributed to the understanding of PDAC pathogenesis and thereby, provided opportunities to characterize and detect disease progression from benign to malignant stages with imaging tools.

### 4.1 PDAC mouse models

With the aim of producing high fidelity, preclinical models representative of the architectural and functional complexity of human PDAC, various mouse models including carcinogen‐induced and genetically engineered murine pancreatic tumor models have been currently employed for preclinical ultrasound imaging researches.

#### 4.1.1 Xenograft models of PDAC

The most commonly employed PDAC animal model consists of the implantation of human cancer cell lines into immuno‐compromised mice or athymic nude mice, either subcutaneously (i.e., heterotopic models) or directly into the pancreas in which the tumor originated (i.e., orthotopic models). Two xenografts models exist: cell line derived xenografts (CDX) and patient derived tumor xenografts (PDX). For a low cost, quick, simple tumor growth monitoring and good reproducibility, heterotopic CDXs are frequently used. Cell lines such as AsPC‐1,^105^, ^115^ BxPC‐3,^47^, ^92^, ^113^ Capan‐1,^117^ Capan‐2,^102^, ^110^, ^120^ COLO‐357,^94^, ^97^ MIA PaCa‐2,^74^, ^106^ Panc‐1^95, 129^ and PL45 cells^108^ are commonly employed for preclinical PDAC molecular imaging. Nevertheless, heterotopic CDXs are considered poorly “realistic”, as tumors arise from the injection of genetically homogeneous cancer cells. Additionally, rapid tumor development lacks many features that are characteristics of human tumor development, notably the chronic inflammatory microenvironment. On the other hand, orthotopic models take advantage of a more realistic pancreatic microenvironment but are more challenging and expensive to produce and are more difficult to image. In addition, the dynamic process of tumor angiogenesis is considerably different in murine tumors. To image pancreatic tumors using Thy1‐targeted microbubbles, Abou‐Elkacem et al., have exploited PDAC cells (AsPC1) co‐injected with MILE SVEN 1 (MS1) mouse vascular endothelial cells stably expressing human Thy1 (MS1_Thy1_).^72^ After midline laparotomy to expose the pancreas, AsPC1 cells along with MS1_Thy1_ cells were co‐injected into the head of pancreas, and resulted in PDAC with a neovasculature expressing human Thy1. Orthotopic xenografts were allowed to grow and MS1_Thy1_ cells were shown to integrate within endothelial lining by immunofluorescence staining of blood vessels.^71^, ^72^ Future applications of this mouse model may provide versatility for in vivo testing of neovasculature‐targeted UCAs in mice by simply replacing the human Thy1 gene by any other human gene relevant to tumor angiogenesis. Compared to the CDX models, PDXs are created by transplanting a piece of patient tumor tissue derived from surgical resection or from tumor biopsies, in immunodeficient mice.^90^ Such tissues can be obtained during EUS‐FNA.^156^ Importantly, PDXs provide realistic heterogeneity of tumor cells and are relevant to drug response studies against human PDAC. Although there are some limitations, particularly related to the host immune environment, PDAC PDXs are the closest currently available model to human pathologies.

#### 4.1.2 Genetically engineered mouse models of PDAC

Genetically engineered mouse models (GEMMs) aim to induce the expression of specific oncogenes (such as activating mutations in Kras proto‐oncogene) and/or the down regulation of tumor suppressor genes (such as p53, INK4A/ARF, and Smad4) associated with human PDAC by genetic recombination. Using a variety of mouse strains, a multitude of genetic backgrounds can be developed, such as the most common model LSL‐*Kras* ^*G12D*/+^; LSL‐Trp53^R172H/+^; *Pdx*‐*1‐Cre* (KPC)^89^, ^124^ and *Pdx*‐*1‐Cre*; LSL‐*Kras* ^*G12D*/+^; Ink4a/Arf^−/−^.^16^, ^71^, ^72^, ^73^, ^104^ Last mentioned model has been utilized in molecular ultrasound imaging due to the expression of Thy1 or VEGFR2 on its tumor neovasculature.^16^, ^73^ Mice lose weight followed by development of ascites, jaundice and then pancreatic tumors with frequent involvement in the duodenum, stomach and/or spleen. The model demonstrates an early and rapid appearance of PanIN lesions and a tumor development time typically three‐fold faster than LSL‐*Kras* ^*G12D*/+^; *Pdx*‐*1‐Cre* alone (2 vs. 6 months).^157^ GEMMs provide a highly heterogeneous tumor microenvironment and contrary to xenografts, tumors exist in the presence of a competent immune system thus improving the ability to examine therapeutic monitoring to immune‐therapies. Moreover, these animal models provide the possibility to follow the disease formation from early stages of PanIN to primary and metastatic tumors (commonly within the abdomen and to the liver, lung, and brain). However, tumor development is slow and colony maintenance is costly.

#### 4.1.3 Carcinogen‐induced PDAC in mice

The administration of carcinogens via a combination of DNA‐damaging agents (7,12‐Dimethylbenz‐[a]anthracene, DMBA and 12‐*O*‐tetradecanoyl‐phorbol‐13‐acetate, TPA) is another way to induce pancreatic cancer in animal models. Mechanistically, tumor initiation is achieved by DMBA administration that generates a point of mutation in Ha‐Ras, an oncogene encoding a protein called H‐RAS primarily involved in the regulating cell division.^158^ Multiple TPA applications subsequently promote tumor growth by deregulating several signaling networks, including the PKC‐Ras/MAPK cascade that mediates proliferation, differentiation and inflammation of Ha‐ras mutated cells.^159^ Using the two‐step DMBA/ TPA strategy, pancreatic cancer formation, from PanINs to PDAC, was followed in rat model by FI and MRI.^96^ Signal intensities in both FI and MRI appeared to correlate with the disease progression and severity although no quantification was performed. As GEMMS, carcinogen‐induced models offer the possibility of exploring the contribution of the immune system to the therapeutic effects of conventional anticancer drugs against PDAC. Nevertheless, it may require considerable time to establish tumors, is less reproducible, and can cause tumors in other tissues.

#### 4.1.4 Rationale for large animal models of PDAC

Mouse models of human PDAC have limited translatability to clinical settings, particularly for the development of interventional technology that requires a human‐sized model. Large animal models that closely mimics the size, anatomy, and physiology of humans would be of great value for translational research. Pigs present such characteristics, and also have good similarity with human regarding genomic, epigenetic, and immunological characteristics. In 2015, the University of Illinois and the National Swine Resource and Research Center engineered a Cre‐inducible swine model (named the “Oncopig”)^160^ which carries an Lox‐Stop‐Lox (LSL)‐cassette containing dominant negative TP53 and activated KRAS (i.e., porcine analog of the KRAS/p53 mouse). Immunodeficient pigs also proved to support the growth of subcutaneously xenografted human pancreatic carcinoma cells (PANC‐1 cells).^161^ Thus, porcine model of pancreatic cancer could enable development of new devices for which murine models have limited utility and can constitute highly predictive preclinical model in which anti‐cancer therapies can be tested and optimized prior to a clinical trial.

### 4.2 Pancreatitis mouse models

Differentiation of PDAC from normal or benign conditions such as pancreatitis, a known risk factor for pancreatic cancer, is important for imaging‐based diagnostic. Development of highly sensitive ultrasound molecular imaging tools requires the ability of contrast agent to target PDAC and not pancreatitis. Chronic pancreatitis is defined as a pathological auto‐digestion of the pancreas and fibro‐inflammatory syndrome that leads to irreversible morphological changes, a progressive loss of endocrine and exocrine functions, and increased risk of PDAC. Among various experimental models of chronic pancreatitis, the most employed models are generated by surgical ligation of the pancreatic duct, alcohol‐induction, repetitive cerulein or l‐arginine injections, and toxic chemical induced models.

Pancreatic duct ligation or intravenous administration of the chemical dibutyltin dichloride (DBTC) are effective ways for inducing pancreatitis that progressively develops chronic features similar to those in human disease.^162^ However, these models are criticized for their lack of reproducibility and thus alternative models are preferred. Alcohol consumption is one of the major etiologic factors predisposing for chronic pancreatitis. Consequently, preclinical trials have been conducted with chronic alcohol administration in animal models. However, alcohol ingestion alone did not induce chronic pancreatitis, despite long experimental durations, and a combination of alcohol and various agents such as cerulein is needed.^163^ Recurrent episodes of acute pancreatitis frequently lead to chronic pancreatic injury in humans. The cerulein‐induced chronic pancreatitis model consists in repeated bouts of cerulein‐induced acute pancreatitis in the course of several weeks causing chronic injury to the pancreas. Using this technique, macrophage accumulation associated with pancreatitis have been successfully imaged by PET using a small‐molecule radiotracer, 125I‐iodo‐DPA‐713, targeting the translocator protein (TSPO).^164^ Imaging results revealed 2.1‐fold higher pancreatic uptake in cerulein‐treated mice compared to control mice. Unfortunately, no uptake comparison has been done on pancreatic cancer models. Finally, the long‐term high dose administration of the essential amino acid l‐arginine caused progressive degeneration of the pancreas. Although of long duration and highly toxic to mice, this experimental model is simple to carry out and shows features similar to those of human chronic pancreatitis. To compare the sensitivity of imaging technologies in differentiating carcinoma from pancreatitis mimicking carcinoma, mouse models of l‐arginine‐induced chronic pancreatitis have also been utilized for contrast agent development.^16^, ^71^, ^72^ in vivo imaging of tumors by ultrasound molecular imaging did not enhance imaging signal from the pancreatitis or normal pancreatic tissues but produced four‐ to eight‐fold higher imaging signal intensity in the human Thy1‐positive orthotopic xenograft tumors and transgenic PDAC mouse models.

Due to difficulties in replicating human chronic pancreatitis in animal models and to respect the current ethical standards regarding animal experimentation that recommends the “three Rs” (replacement, reduction, and refinement), as well as for practical reasons, alternative in vitro techniques are worth consideration. They include organ on a chip, in silico models or 3D bio‐printing. For a thorough discussion, we refer our readers to the review published by Swayden et al.^165^

## 5 CLINICAL TRANSLATION OF PDAC MOLECULAR IMAGING

### 5.1 PDAC heterogeneity and molecular sub‐typing

The majority of pancreatic cancer arises from microscopic precursor lesions, named pancreatic intraepithelial neoplasia (PanIN), currently undetectable with clinical imaging modalities. Some cases can also arise from macroscopic cystic lesions of the pancreas such as intraductal papillary mucinous neoplasms (IPMNs) and mucinous cystic neoplasms (MCNs).^3^ PDAC molecular pathology is dominated by the oncogenic mutation of KRAS (>90% of cases). Frequently altered tumor suppressor genes include the inactivation of TP53 (60–70%), CDKN2A (40–50%), and SMAD4 (30–40%).^3^ One major issue of PDAC is the high degree of heterogeneity observed between patients regarding PDAC origin, symptoms, clinical evolution, predisposition to early metastasis, and sensitivity to treatments, in addition to the heterogeneity observed within the same tumor. Molecular sub‐typing of PDAC is needed to improve patient selection for specific treatments, and to focus diagnostic and therapeutic strategies on potential biomarkers adequate with the patient sub‐type. Other genomic attributes such as the structural variation of the genome can be used to group pancreatic tumors. Molecular sub‐typing for pancreatic cancer is in its infancy, yet PDAC can be classified into four categories: stable genomes (<50 structural variants per genome); scattered genomes (50–200 structural variants per genome); locally rearranged genomes (>200 structural variants clustered on <3 chromosomes); or unstable genomes (>200 structural variants distributed across the genome).^166^ Thus, given the heterogeneous nature of PDAC in patients, use of an universal biomarker may not be realistic, and therefore the potential to perform multiplexing is crucial. In PDAC, a large number of biomarkers are known to be overexpressed. However, a limited number of these markers are eligible candidates for vascular targeted imaging. Biomarker expression must be exclusive of chronic pancreatitis, homogeneous through tumor tissue, and have significant upregulation relative to normal and surrounding tissue. The ideal early detection diagnostic strategy will likely use multiple biomarkers with confirmatory real‐time molecular imaging.

### 5.2 Clinical trials using targeted UCAs

Transabdominal ultrasound is the only imaging technology to be at the same time relatively nonexpensive, available, noninvasive and without ionizing radiation. Moreover, some patients cannot undergo MRI or PET/CT due to contraindications, thus reinforcing the importance of US imaging in the clinic. Latest advances in both molecularly targeted microbubble composition, structure, and contrast specific ultrasound techniques could improve the quality and accuracy of PDAC diagnosis and staging in the population. The first and currently only clinical grade molecularly targeted UCA, named BR55 (Bracco, Geneva, Switzerland), has been moved into multiple clinical trials in Europe and in the USA. This contrast agent is composed of a phospholipid shell of 1.5 μm mean diameter, with perfluorobutane/nitrogen gas in the core. BR55 is targeted against the kinase insert domain receptor (KDR), the human analog of vascular endothelial growth factor type 2, VEGFR2, which is well documented for its vasculature overexpression in various cancer types.^73^, ^167^ BR55 uses a KDR targeted heterodimer peptide directly incorporated into the microbubble shell via the amino group of DSPE‐PEG2000‐ NH2. An early phase 1 clinical trial was performed using BR55 to identify area of VEGFR2 expression in prostate cancer (ClinicalTrials.gov identifier: NCT01253213), and successful results stimulated further developments. A year after, BR55 entered phase 2 trials to evaluate its sensitivity and specificity for prostate cancer detection (ClinicalTrials.gov identifier: NCT02142608). Recently, two phase 2 clinical trials have been started for the characterization of ovarian lesions (ClinicalTrials.gov identifiers: NCT04248153 and NCT03493464) while another phase 2 trial has been started for the characterization of pancreatic lesions in patients with suspected PDAC after transabdominal ultrasound (ClinicalTrials.gov identifier: NCT03486327). Such microbubble technology can have multiple potential in clinical applications, including imaging inflammation, ischemia, atherosclerosis, and cancers of various types, by choosing an ideal targeting ligand for the specific biomarker. For insight on clinical translation of ultrasound molecular imaging using microbubbles, we refer our readers to the publication by Abou‐Elkacem et al.^77^ Although targeted CEUS exhibits high sensitivity, molecular targets are currently constrained to the vascular lumen as the microbubbles are unable to extravasate from the vessels into the interstitium owing to their larger size. The use of other imaging modalities, like PET/CT, can thus bring additional, rather than competitive, information by using probes able to extravasate the vasculature.

### 5.3 Clinical trials using other molecular imaging modalities

Multiple other clinical trials on pancreatic cancer molecular imaging have been designed with different imaging modalities. Notably, integrin alpha‐v‐beta peptide‐radiotracers were studied for scanning patients using PET/CT (Early phase 1, Drug: [18F]‐R01‐MG‐F2, ClinicalTrials.gov identifier: NCT02683824),^47^ (Drug: 68Ga‐cycratide, approved by the Institutional Review Board of Peking University Cancer Hospital et Institute, #2018KT54).^48^ In addition, CA19‐9 antibody‐based radiotracers have been tested in a dose‐escalation trial (Phase 1, Drugs: MVT‐2163 and MVT‐5873, ClinicalTrials.gov identifier: NCT02687230).^123^ On the other hand, fluorescence imaging is very frequent in preclinical research but a major drawback for clinical application of optical techniques originates from the low tissue‐penetration as fluorescent dyes can only be detected superficially (up to 1 cm depth).^168^ This limitation prevents the use of optical imaging when no direct access to the organ is available, as is the case in most clinical presentations. However, such techniques can be implemented to help surgeons during tumor resection procedures using fluorescent laparoscopy. Multiple trials have employed specific antibodies conjugated with a NIR (Near Infra‐Red) emitting fluorochrome (Phase 2, Drug: SGM‐101, ClinicalTrials.gov identifier: NCT02973672),^109^ (Phase 1/2, Drugs: Panitumumab‐IRDye800 and Cetuximab‐IRDye 800, ClinicalTrials.gov identifiers: NCT02736578 et NCT03384238).^114^, ^130^

### 5.4 Challenges of patient recruitment in clinical trials

Stratification of patients with diagnosed PDAC in small cohorts based on screening (e.g., presence of a particular biomarker, genomic mutations, or structural variation of the genome) may allow better clinical results and could certainly benefit the design and development of new drugs. Importantly, patient recruitment for clinical trials remains a major barrier to rapid execution of diagnosis and drug development programs. Statistically relevant results necessitate a maximized number of patients in a given clinical trial. However, the heterogenous nature of PDAC, its low prevalence in the population, the under‐diagnosed patient number and its severity, diminish potential patient enrollment.

## 6 CONCLUSION

Pancreatic cancer is difficult to diagnose at early stages with conventional clinical imaging tools. Present imaging methods are impaired by the ambiguous or nonrelevant imaging results to accurately detect PDAC in high risk patient groups. We discussed in this review the unique properties of microbubbles as true intravascular probes with proven value as tissue contrast agents for ultrasound molecular imaging of PDAC. CEUS using vascular‐targeted microbubbles offer a noninvasive method to diagnose tumor angiogenesis. Supported by the success of the first targeted‐microbubble entering clinical trial, BR55, and by favorable preclinical results, the clinically compatible microbubble targeting the vascular protein Thy1 is of outstanding interest. Importantly, its ability to discriminate between pancreatitis and PDAC in mouse models should certainly provide opportunities for improved PDAC prognosis.

### 1 CONFLICT OF INTEREST

The authors declare no conflict of interest.

**ABBREVIATIONS**
CA 19‐9: serum carbohydrate antigen 19‐9
CDX: cell line derived xenograft
CE‐EUS: contrast‐enhanced endoscopic ultrasound
CEST: chemical exchange saturation transfer
CETUS: contrast‐enhanced targeted ultrasound
CEUS: contrast‐enhanced ultrasound
CT: computed tomography
DBTC: dibutyltin dichloride
DMBA: 7,12‐dimethylbenz[a]anthracene
ECM: extracellular matrix
ELIP: echogenic liposome
EPR: enhanced permeability and retention
EUS: endoscopic ultrasound
FA: folic acid
FDA: food and drug administration
FI: fluorescence imaging
FNA: fine needle aspiration
GEMM: genetically engineered mouse model
GV: gas vesicle
IPMN: intraductal papillary mutinous neoplasm
KDR: kinase insert domain receptor
MB: microbubble
MCN: mutinous cystic neoplasm
MDCT: multi‐detector computed tomography
MMP: matrix metalloproteinase
MRCP: magnetic resonance cholangiopancreatography
MRI: magnetic resonance imaging
NB: nanobubble
NHS: *N*‐hydroxysuccinimide
PanIN: pancreatic intraepithelial neoplasia
PCD: phase‐change droplet
PDAC: pancreatic ductal adenocarcinoma
PDX: patient derived tumor xenografts
PEG: polyethylene glycol
PEPT1: peptide transporter 1
PET: positron emission tomography
PSMA: prostate specific membrane antigen
ScFv: single‐chain antibody fragment
Thy1: thymocyte differentiation antigen
TPA: 12‐*O*‐tetradecanoyl‐phorbol‐13‐acetate
TSPO: translocator protein
UCA: ultrasound contrast agent
uPA: urokinase‐type plasminogen activator
uPAR: urokinase‐type plasminogen activator receptor
US: ultrasound
18F‐FDG: 18F‐fluoro‐2‐deoxy‐D‐glucose
